# The lysosomal proteome of senescent cells contributes to the senescence secretome

**DOI:** 10.1111/acel.13707

**Published:** 2022-09-10

**Authors:** Miguel Rovira, Rebecca Sereda, David Pladevall‐Morera, Valentina Ramponi, Ines Marin, Mate Maus, Julio Madrigal‐Matute, Antonio Díaz, Fernando García, Javier Muñoz, Ana María Cuervo, Manuel Serrano

**Affiliations:** ^1^ Cellular Plasticity and Disease Group Institute for Research in Biomedicine (IRB Barcelona), Barcelona Institute of Science and Technology (BIST) Barcelona Spain; ^2^ Department of Developmental and Molecular Biology Albert Einstein College of Medicine New York New York USA; ^3^ Institute for Aging Studies Albert Einstein College of Medicine New York New York USA; ^4^ Department of Cellular and Molecular Medicine, Center for Chromosome Stability and Center for Healthy Aging University of Copenhagen Copenhagen Denmark; ^5^ Instituto Biomédico de Nutrición y Salud Elda Spain; ^6^ Proteomics Unit Spanish National Cancer Research Center (CNIO) Madrid Spain; ^7^ Biocruces Bizkaia Health Research Institute Barakaldo Spain; ^8^ Ikerbasque, Basque Foundation for Science Bilbao Spain; ^9^ Catalan Institution for Research and Advanced Studies (ICREA) Barcelona Spain

**Keywords:** aging, autophagy, cellular senescence, exocytosis, lysosome, SASP

## Abstract

Senescent cells accumulate in tissues over time, favoring the onset and progression of multiple age‐related diseases. Senescent cells present a remarkable increase in lysosomal mass and elevated autophagic activity. Here, we report that two main autophagic pathways macroautophagy (MA) and chaperone‐mediated autophagy (CMA) are constitutively upregulated in senescent cells. Proteomic analyses of the subpopulations of lysosomes preferentially engaged in each of these types of autophagy revealed profound quantitative and qualitative changes in senescent cells, affecting both lysosomal resident proteins and cargo proteins delivered to lysosomes for degradation. These studies have led us to identify resident lysosomal proteins that are highly augmented in senescent cells and can be used as novel markers of senescence, such as arylsulfatase ARSA. The abundant secretome of senescent cells, known as SASP, is considered their main pathological mediator; however, little is known about the mechanisms of SASP secretion. Some secretory cells, including melanocytes, use the small GTPase RAB27A to perform lysosomal secretion. We found that this process is exacerbated in the case of senescent melanoma cells, as revealed by the exposure of lysosomal membrane integral proteins LAMP1 and LAMP2 in their plasma membrane. Interestingly, a subset of SASP components, including cytokines CCL2, CCL3, CXCL12, cathepsin CTSD, or the protease inhibitor SERPINE1, are secreted in a RAB27A‐dependent manner in senescent melanoma cells. Finally, proteins previously identified as plasma biomarkers of aging are highly enriched in the lysosomes of senescent cells, including CTSD. We conclude that the lysosomal proteome of senescent cells is profoundly reconfigured, and that some senescent cells can be highly active in lysosomal exocytosis.

## Graphical abstract

Here, we report that senescent cells upregulate the two major types of autophagy, chaperone‐mediated autophagy, and macroautophagy. We analyze the proteome of lysosomes engaged in each pathway, identifying novel markers of senescent cells. We also report that some senescent cells can perform direct lysosomal exocytosis by which lysosomal components contribute to the SASP and to serum markers of aging.

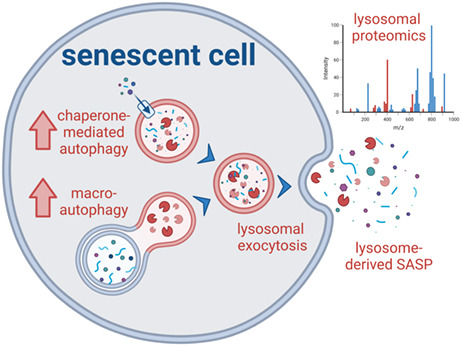

## 1 INTRODUCTION

Lysosomes are membrane‐bound intracellular organelles with a relevant role in metabolism and in organelle and protein quality control (Lawrence & Zoncu, 2019). These organelles are characterized by a single limiting membrane and an acidic lumen enriched in resident hydrolases, including numerous proteases. Lysosomes mediate the degradation and recycling of intracellular components delivered to lysosomes through autophagy, and extracellular material captured in endosomes and phagosomes (Levine & Kroemer, 2008; Settembre et al., 2013; Xu & Ren, 2015). Lysosomes also perform other important cellular functions such as Ca^2+^‐buffering, intracellular signaling, and direct extracellular secretion of the lysosomal contents. The latter process, known as lysosomal secretion, is a Ca^2+^‐dependent mechanism active in osteoclasts, melanocytes, endothelial cells, and cells from the hematopoietic lineage, including lymphocytes, neutrophils, mast cells, and macrophages (Luzio et al., 2014; Schmidt et al., 2009; Settembre et al., 2013; Sheshachalam et al., 2014).

It has long been known that the lysosomal compartment is largely expanded in senescent cells (Robbins et al., 1970). In fact, the characteristic senescence‐associated β‐galactosidase (SAβGal) activity detected in senescent cells reflects the increased lysosomal mass of senescent cells (Dimri et al., 1995; Kurz et al., 2000; Lee et al., 2006). Similarly, most tested lysosomal hydrolases are also enriched in senescent cells, including α‐mannosidase, α‐fucosidase, and N‐acetyl‐β‐hexosaminidase (Knaś et al., 2012). However, there is a lack of comprehensive information on the proteome of lysosomes of senescent cells, including both lysosome‐resident and cargo proteins.

The intracellular proteins degraded in lysosomes in mammalian cells generally enter through two separate routes, macroautophagy and chaperone‐mediated autophagy. Macroautophagy (MA) is responsible for capturing multiprotein complexes, such as ribosomes, protein aggregates, and organelles, inside double‐membrane vesicles (autophagosomes) that deliver their cargo to lysosomes through vesicular fusion. MA is potentiated during cellular senescence, and its inhibition delays the establishment of senescence and the senescence‐associated secretory phenotype (SASP) (Gamerdinger et al., 2009; Young et al., 2009). Interestingly, prolonged activation of MA during senescence leads to the activation of mTOR, thereby facilitating protein synthesis and contributing to the SASP (Herranz et al., 2015; Laberge et al., 2015; Narita et al., 2011). Chaperone‐mediated autophagy (CMA) constitutes another important mechanism of intracellular protein degradation in lysosomes through direct funneling of protein cargoes across the lysosomal membrane. Substrate proteins are targeted by the chaperone HSC70 to lysosomes and internalized through a translocation complex formed by the lysosomal membrane protein LAMP2A (Kaushik & Cuervo, 2018). Despite the important roles described for CMA in the maintenance of the metastable proteome and in the regulation of cellular processes such as metabolism, cell cycle, transcription, or cell death (Bourdenx et al., 2021; Park et al., 2015; Schneider et al., 2014; Valdor et al., 2014), little is known about CMA in senescent cells.

Here, we have characterized the autophagic flux through MA and CMA in senescent cells, observing a previously unknown increase in CMA activity, which is parallel to the known increase in MA, albeit with different kinetics. Taking advantage of the possibility of isolating lysosomal subpopulations preferentially engaged in MA or CMA, we present here a comprehensive quantitative proteomic analysis of purified lysosomes from senescent cells, which reveals selective changes in the composition and quantity of resident proteins and cargo proteins undergoing lysosomal degradation. Interestingly, we also found that lysosomal secretion contributes to the SASP in a RAB27A‐dependent manner in melanoma senescent cells. These data constitute a useful resource to further elucidate the interplay between autophagy and senescence, and to find new biomarkers and vulnerabilities in senescent cells.

## 2 RESULTS

### 2.1 Increased lysosomal biogenesis in senescent cells

**FIGURE 1:**
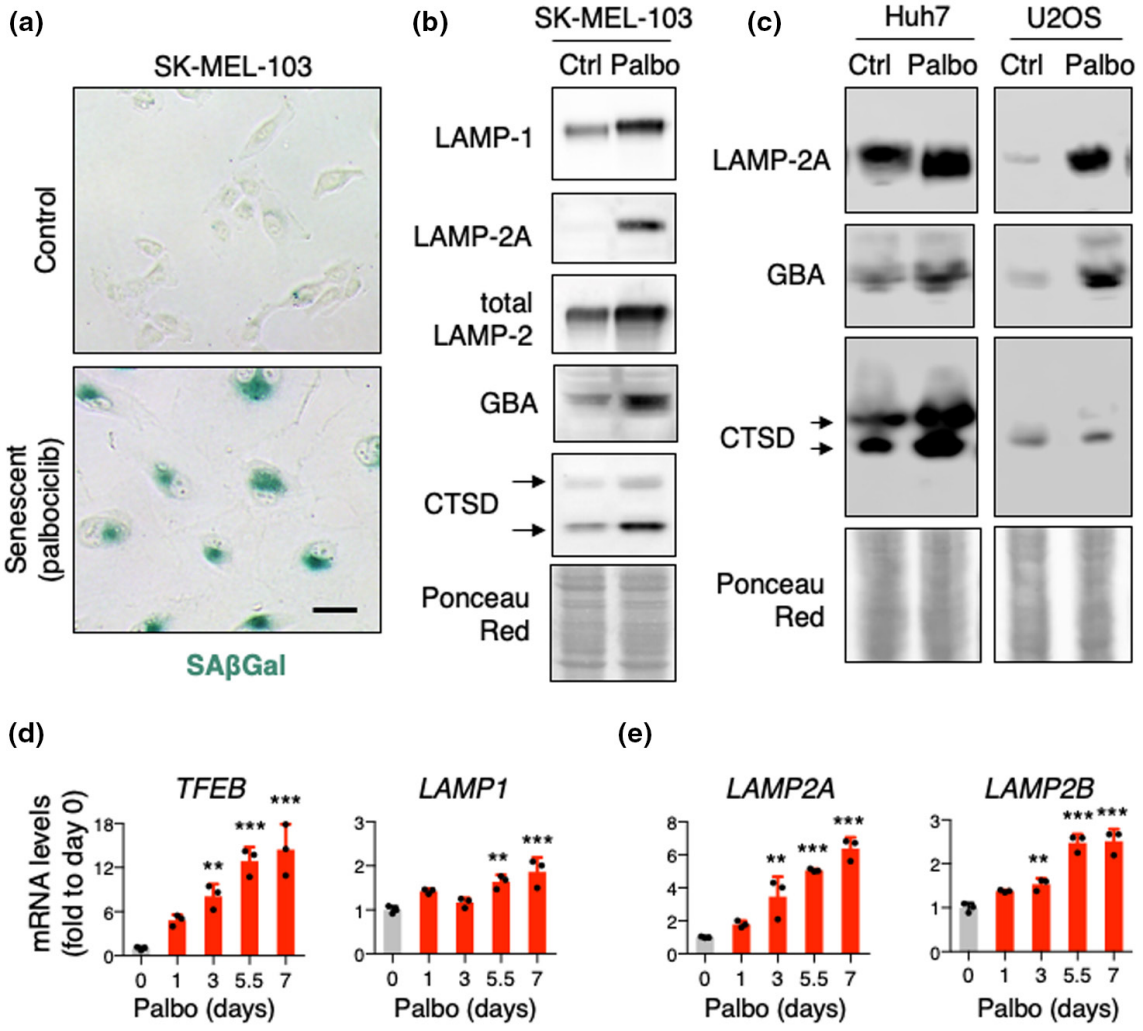
Characterization of the lysosomal system in senescence. (a) Representative SAβGal staining pictures of SK‐MEL‐103 cells treated with 2 μM palbociclib for 1 week. Scale bar 50 μM. (b) Western blot analysis for LAMP1, LAMP2A, LAMP2, GBA, and CTSD in SK‐MEL‐103 cells. Arrows indicate precursor and mature CTSD. Ponceau staining is shown as loading control. (c) Western blot analysis for LAMP2A, GBA, and CTSD in Huh7 and U2OS cells treated with 2 μM palbociclib for 1 week. Ponceau staining is shown as loading control. (d, e) mRNA levels of *TFEB*, *LAMP1* (d) and *LAMP2A*, *LAMP2B* (e) in SK‐MEL‐103 cells treated with 2 μM palbociclib at the indicated timepoints. *ACTB*, *18S rRNA*, and *B2M* were used for input normalization (mean of the three housekeepers). Values are relative to control cells and are expressed as mean ± SD, and statistical significance was assessed by one‐way ANOVA and Dunnett's multiple comparisons test (versus control group). *p < 0.05

To study lysosomal function in senescent cells, we began by confirming the expansion of the lysosomal compartment in cellular models of senescence. Melanoma SK‐MEL‐103 cells treated with the CDK4/6 inhibitor, palbociclib, displayed SAβGal staining and increased protein levels of the lysosomal membrane proteins LAMP1 and LAMP2 (Figure 1a). This increase was particularly pronounced in the case of LAMP2A, the only spliced variant of the *Lamp2* gene required for CMA and a limiting component for this type of autophagy (Cuervo & Dice, 2000a). Luminal lysosomal hydrolases, such as β‐glucocerebrosidase (GBA) and cathepsin D (CTSD), were also increased in senescent SK‐MEL‐103 cells (Figure 1b). Similar findings were observed in hepatocarcinoma Huh7 and osteosarcoma U2OS cells treated with palbociclib (Figure 1c). To ascertain whether the expansion of lysosomes in senescence was accompanied by de novo lysosomal synthesis, we analyzed the expression of the transcription factor EB (TFEB), the master regulator of lysosomal biogenesis (Napolitano & Ballabio, 2016). Interestingly, *TFEB* mRNA levels dramatically increased over time after palbociclib addition, preceding an elevation in LAMP1 known to be part of the TFEB‐transcriptional program (Figure 1d). We also found marked elevation of lysosomal components that are not under TFEB regulation, such as *LAMP2A* and *LAMP2B* (Figure 1e). Of note, transcriptional upregulation of the LAMP2A variant (~sixfold), which acts as a receptor for CMA (Cuervo & Dice, 2000b), was higher than the upregulation of LAMP2B (~2.5‐fold), which does not play a specific role in CMA (Cuervo & Dice, 2000b) (Figure 1e). Together, these results indicate that senescent cells enlarge the lysosomal compartment due, at least in part, to de novo biogenesis of lysosomes.

### 2.2 Macroautophagy (MA) is increased in senescent cells

**FIGURE 2:**
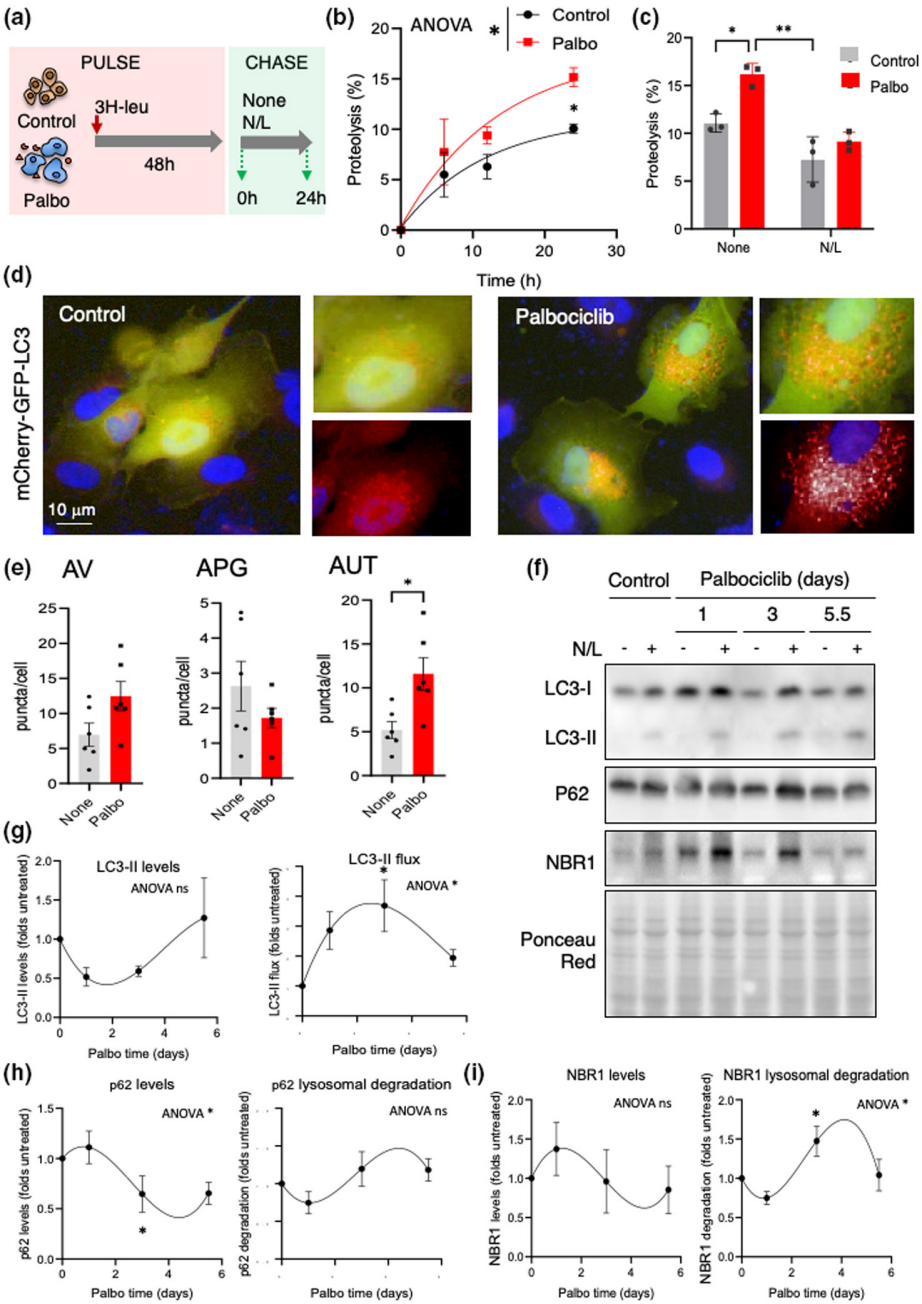
Upregulation of MA in senescent cells. (a) Scheme of the assay to determine proteolytic rates of long‐lived proteins in control and palbociclib‐treated cells and analysis of lysosomal contribution to degradation. (b) Protein degradation rates of long‐lived proteins in control and 7 days palbociclib‐treated SK‐MEL‐103 cells. Values are mean ± SEM and are expressed as percentage proteolysis. *n* = 3 in two different experiments. Statistical significance was assessed by two‐way ANOVA and Sidak's multiple comparisons test. Differences among time points were significant for *p* < 0.0001 and between control and palbociclib‐treated cells (shown in the legend) for *p* < 0.05. (c) Percentage of proteolysis of long‐lived proteins in the cells in (b) after 24 h of culture without additions (None) or in presence of ammonium chloride and leupeptine (N/L). Statistical significance was assessed by two‐way ANOVA and Tukey's multiple comparisons test (versus control group). Differences between control and palbociclib‐treated cells and between None and N/L are shown in the figure. (d) Representative images of control and 7 days palbociclib‐treated SK‐MEL‐103 cells stably transduced with a lentivirus expressing the tandem reporter mCherry‐GFP‐LC3 to monitor autophagic flux. Insets: higher magnification of merged channels or red channel. Nuclei are highlighted with DAPI. (e) Quantification of autophagic vacuoles (AV, combination of autophagosomes (APG) and autolysosomes (AUT)) (left), APG (mCherry^+^ GFP^+^ vesicles) (middle) and AUT (mCherry^+^ GFP^−^ vesicles) (right) in cells as in (d. Values are expressed as puncta per cell section and are individual values and mean ± SEM. *n* = 6 experiments with >1200 cells/condition. Statistical significance was assessed by the two‐tailed Student's *t*‐test (versus control group). (f–i) Representative immunoblot for the indicated proteins (f) and densitometric quantification of steady‐state values (left) or lysosomal degradation for LC3‐II (g), p62 (h), and NBR1 (i) in SK‐MEL‐103 cells at the indicated days after addition of palbociclib (2 μM) to the media. Cells were supplemented with ammonium chloride and leupeptin (N/L) for 4 h where indicated (+). Ponceau red staining is shown as loading control. Quantifications are shown as folds of the cells no supplemented with palbociclib (Control) and values are mean ± SEM (*n* = 3 experiments). Statistical significance was assessed by the one‐way ANOVA and Tukey's multiple comparisons test (versus control untreated). Significant differences by ANOVA are indicated in the graph and differences with time 0 at specific time points in the graph.

Next, we wondered whether the expanded lysosomal compartment in senescent cells was indeed functional. In general, proteins degraded in lysosomes have long half‐lives (Dice, 1987), and metabolic labeling pulse and chase experiments in cultured cells, using radiolabeled amino acids and lysosomal inhibitors, can be used as a good assessment of lysosomal degradative function (Kaushik & Cuervo, 2009). Thus, to study the lysosomal degradative capacity of senescent cells, we first treated SK‐MEL‐103 cells with palbociclib to induce senescence and then added ^3^H‐leucine to the media for 48 h to radiolabel de novo synthesized proteins (pulse period). After extensive washing, we measured the breakdown of radiolabeled proteins (chase period) as the release of free ^3^H‐leucine into the culture medium (Figure 2a). We found a significant increase in the degradation rate of long‐lived proteins in senescent cells compared to their non‐senescent counterparts. Moreover, these differences were ablated upon addition of inhibitors of lysosomal proteolysis consisting of ammonium chloride (NH_4_Cl), a weak base that neutralizes the lysosomal acidic pH required by many lysosomal enzymes, and leupeptin, a potent inhibitor of cysteine and serine peptidases (the two components abbreviated as N/L) (Figure 2b,c). Therefore, we conclude that the higher rates of protein degradation in senescent cells mostly reflect their increased degradation in lysosomes.

Previous studies have reported increased levels of macroautophagy (MA) in replicative senescence and in oncogene‐induced senescence (Gamerdinger et al., 2009; Narita et al., 2011; Young et al., 2009). We monitored MA in SK‐MEL‐103 cells using a tandem fluorescent LC3 construct (mCherry‐GFP‐LC3) (Kimura et al., 2007). This reporter detects autophagosomes (APG) as puncta positive for both mCherry and GFP fluorophores. Upon fusion of APGs with lysosomes, the resulting autolysosomes (AUT) are detected as puncta positive only for mCherry because GFP fluorescence is quenched by the acidic lysosomal pH. Cells were stably transduced with mCherry‐GFP‐LC3 and were left untreated or treated with palbociclib for 7 days to induce senescence. We observed a significantly higher number of AUT in senescent SK‐MEL‐103 cells, indicating higher levels of MA flux (Figure 2d,e). We noted a trend toward higher overall content of autophagic vacuoles (AV) (APG plus AUT), but lower abundance of APG in senescent cells (Figure 2e). This is compatible with increased autophagic flux resulting from both higher APG formation and accelerated APG maturation. To gain insights on the time course of MA changes during palbociclib‐induced senescence and to compare *in bulk* and selective MA, we next analyzed the flux of LC3‐II, as well as lysosomal degradation of p62 and NBR1, two well‐known receptors for selective MA, at different times after palbociclib administration (Figure 2f–h). Lysosomal degradation was calculated as the increase in the cellular levels of these proteins (as fold change) after preventing their proteolysis inside lysosomes by treating the cells with NH_4_Cl and leupeptin (N/L). We found that exposure to palbociclib led to a rapid upregulation of MA (up to fourfold increase in LC3‐II flux at Day 3), followed by a gradual decrease until reaching approximately a twofold increase in LC3‐II flux by Day 5.5 (Figure 2f,g right) similar to the increase observed after that day with the fluorescent reporter (Figure 2d,e). As expected, steady‐state levels of LC3‐II followed an inverse pattern to the LC3‐II flux (Figure 2f,g left). Interestingly, lysosomal degradation of the two selective MA receptors, p62 and NBR1, followed different kinetics, with their peak of degradation and lower steady‐state levels toward the later stage of senescence establishment (Figure 2f,h,i). This further confirms that both *in bulk* and selective (p62‐ or NBR1‐dependent) MA are upregulated in senescent cells, with *in bulk* MA preceding the activation of selective MA.

### 2.3 Chaperone‐mediated autophagy is increased in senescent cells

**FIGURE 3:**
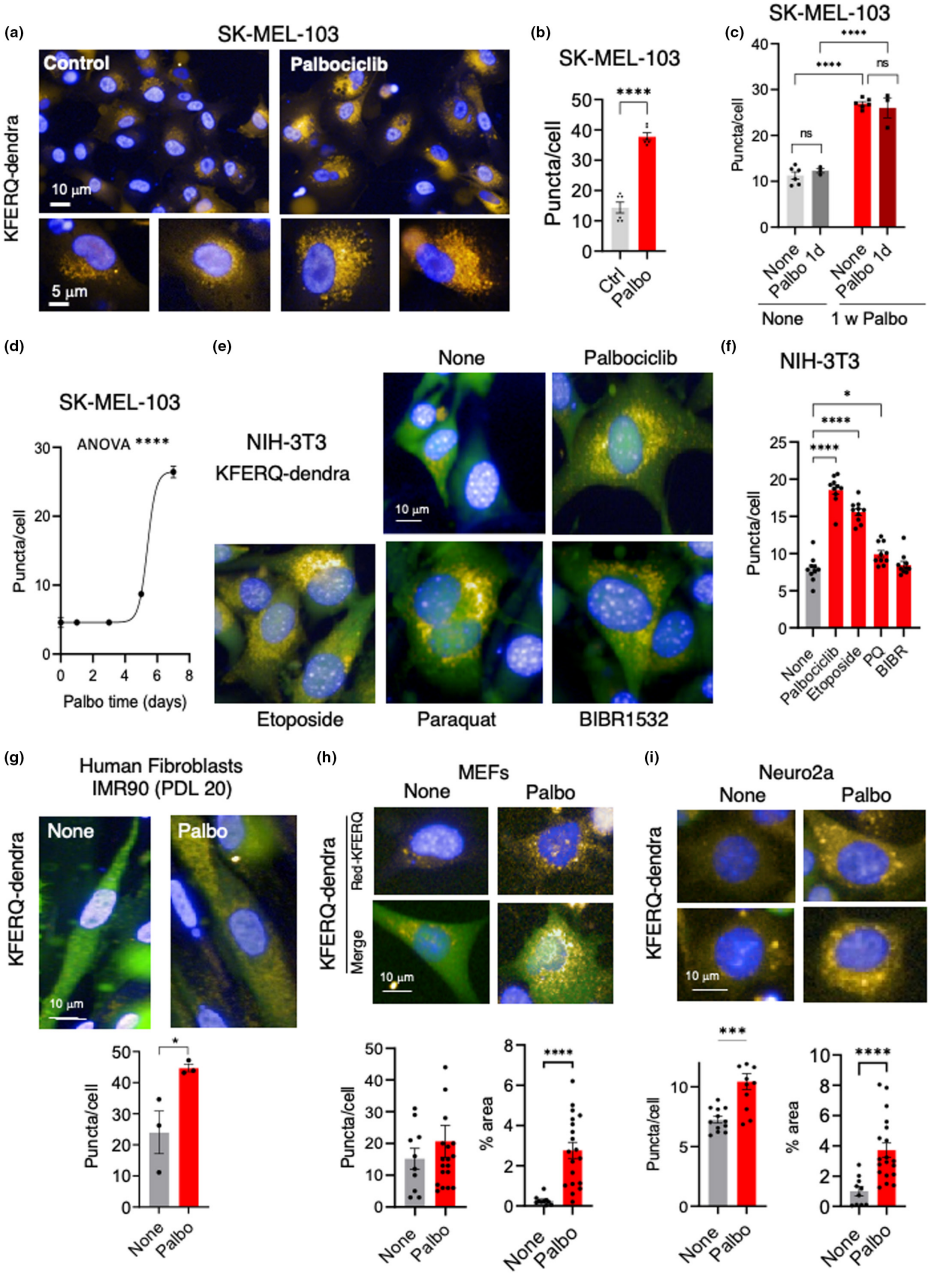
Upregulation of CMA in senescent cells. (a) Representative images of control and 7 days palbociclib‐treated SK‐MEL‐103 cells stably transduced with a lentivirus expressing a KFERQ‐dendra reporter to monitor CMA activity. Cells were photoswitched, and fluorescence in the red channel (pseudocolored in yellow, in consideration for color‐blind readers) was monitored using high‐content miscroscopy. Insets: higher magnification of red channel images. Nuclei are highlighted with DAPI. (b) Quantification of changes in the mean number of puncta per cell section quantified with high‐content microscopy. All values are individual values and mean ± SEM of six separate wells for each condition, and quantifications were done in at least 2500 cells per condition. Statistical significance was assessed by the two‐tailed Student's *t*‐test. (c) Quantification of changes in the mean number of puncta per cell section of control and 7 days palbociclib‐treated SK‐MEL‐103 cells 1 day after the addition of a single doses of palbociclib. All values are individual values and mean ± SEM of six separate wells for each condition, and quantifications were done in at least 2500 cells per condition. Statistical significance was assessed by the two‐way ANOVA and Sidak's multiple comparisons test (versus control group). (d) Time course of CMA activity calculated in SK‐MEL‐103 cells expressing the KFERQ‐dendra reporter at the indicated times after addition of pablobiclib. Values are expressed as puncta per cell section and are mean ± SEM of three separate wells for each condition, and quantifications were done in >2500 cells per condition. Statistical significance was assessed by the one‐way ANOVA and Dunnett's multiple comparisons test. (e) Representative images of NIH 3T3 cells stably transduced with a lentivirus expressing a KFERQ‐dendra reporter to monitor CMA activity 7 days after the indicated treatments. Cells were photoswitched and imaged with high‐content microscopy as in (a). Merged images of fluorescence in the green and red (pseudocolored in yellow) channels are shown. Nuclei are highlighted with DAPI. (f) Quantification of changes in the mean number of puncta per cell section in cells in (e). All values are individual values and mean ± SEM. Quantifications were done in >600 cells/condition in 3 independent experiments. Statistical significance was assessed by the one‐way ANOVA and Dunnett's multiple comparisons test. (g) Representative images of primary human fibroblasts (IMR90 cells at population doubling level (PDL) 20) transduced with a lentivirus expressing a KFERQ‐dendra reporter to monitor CMA activity 7 days after exposure to palbociclib. Cells were photoswitched and imaged by high‐content microscopy. *Bottom*: Quantification of changes in the mean number of fluorescent puncta per cell. All values are mean ± SEM and individual values of >50 cells/condition in 3 independent experiments. Statistical significance was assessed by the unpaired *t*‐test. (h) Similar studies as in (g) but using primary mouse embryonic fibroblasts (MEFs) and high‐content microscopy. Red channel (top) and merged green and red channels (bottom) are shown. *Bottom*: Quantification of changes in the mean number of fluorescent puncta per cell (left) or in the percentage of cellular area occupied by fluorescent puncta (right). All values are mean ± SEM and individual values. Quantifications were done in >100 cells/condition in 10 independent wells. Statistical significance was assessed by the unpaired *t*‐test. (i) Similar studies as in (g) but using a neuroblastoma cell line. Examples of two cells for each condition in the red channel (pseudocolored in yellow) are shown. *Bottom*: Quantification as in (h) was done in >1000 cells/condition in 10 independent wells. Statistical significance was assessed by the unpaired *t*‐test. ****p < 0.0001, ***p < 0.001, **p < 0.01, *p < 0.05.

Induction of chaperone‐mediated autophagy (CMA) can be monitored with a KFERQ‐photo‐switchable (PS)‐Dendra fluorescent reporter as an increase in fluorescent puncta when this reporter is delivered to lysosomes via CMA (Caballero et al., 2018; Dong et al., 2020, 2021; Juste et al., 2021; Koga et al., 2011). Photoswitching of Dendra fluorescence from green to red allows to distinguish the photoconverted reporter (red, pseudocolored here in yellow, in consideration to color‐blind readers) as it is delivered to CMA lysosomes over the background cytosolic signal of the newly synthesized reporter (green). Transduction of SK‐MEL‐103 cells with this reporter revealed a significant increase in the number of fluorescent puncta in senescent cells (Figure 3a,b). We confirmed that the increase in CMA was not related directly to the presence of palbociclib, as addition of the drug for 1 day to control cells or re‐addition of palbociclib for 1 day to already senescent cells did not upregulate CMA activity (Figure 3c). These results support that CMA upregulation was not a direct consequence of CDK4/6 inhibition but rather a feature of senescence. In fact, a time‐course analysis revealed that upregulation of CMA occurs after the observed increase of *in bulk* MA and coincides temporally with the upregulation of selective MA (Figure 3d compare with Figure 2g–i), thus supporting a switch toward selectivity on lysosomal degradation during senescence.

Since this is the first time that increased CMA has been reported in senescent cells, we examined four additional cell types, namely immortalized mouse fibroblasts (NIH 3T3) (Figure 3e,f), primary human fibroblasts (IMR90 early passage) (Figure 3g), primary mouse embryo fibroblasts (MEFs) (Figure 3h), and mouse neuroblastoma cells (Neuro2a) (Figure 3i). Senescence was induced for 7 days in all these cells by treatment with palbociclib and scored by the loss of HMGB1 (Davalos et al., 2013) (Figure S1A–D). Similar to senescent SK‐MEL‐103 cells, these four additional senescent cell types displayed significantly elevated levels of CMA measured by KFERQ‐Dendra (Figure 3e–i; note that in instances such as MEFs and Neuro2a cells, where lysosomal clustering in the perinuclear area provides difficulty in identification of individual lysosomes, we have also included the analysis of the percentage of cellular area occupied by KFERQ‐Dendra^+^ puncta as an additional read out of upregulated CMA in senescent cells). Furthermore, to investigate whether upregulation of CMA was also observed upon inducing senescence by other stimuli, we treated NIH 3T3 cells with three additional agents, namely etoposide, paraquat and the inhibitor of telomerase, BIBR1532, and 7 days after addition of the stimuli, we observed upregulation of CMA (Figure 3e,f) that was proportional to the level of senescence (scored as loss of HMGB1; Figure S1A) induced by each of the treatments. Therefore, we conclude that senescent cells upregulate CMA degradative pathways.

### 2.4 Proteomics of senescent lysosomes

**FIGURE 4:**
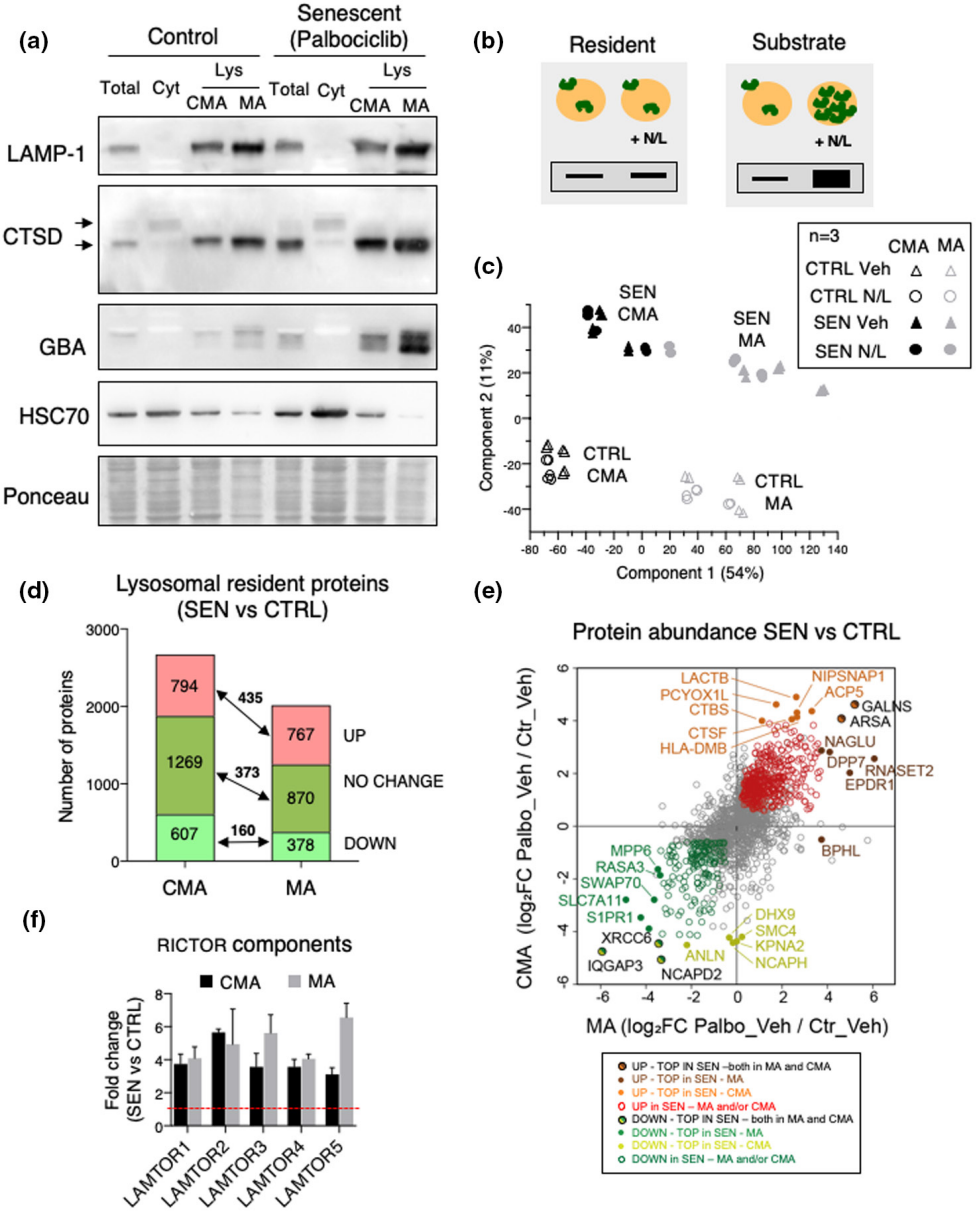
Lysosomal isolation and mass spectrometry analysis of constitutive resident proteins. (a) Western blot analysis for LAMP1, LAMP2A, CTSD, GBA, and HSC70 in homogenate (Total), cytosol (Cyt), and lysosomes (Lys) with high CMA activity (CMA) or high MA activity (MA) isolated from control or palbociclib‐treated SK‐MEL‐103 cells. Arrows indicate precursor and mature form. Ponceau staining is shown as loading control. (b) Scheme of the hypothetical changes in levels of proteins in lysosomes treated or not with ammonium chloride and leupeptin (N/L) 16 h before isolation. Proteins are classified depending on these changes as degraded (lysosomal substrates) if they accumulate upon N/L treatment, or constitutive (resident proteins) if they do not accumulate upon N/L treatment. (c) Principal component analysis (PCA) of the CMA and MA lysosome samples, *n* = 3 with two technical replicates each. (d) Changes in abundance of the lysosomal constitutive proteins in CMA and MA lysosomes. Constitutive proteins were defined as those that do not change significantly *p* > 0.05 in N/L versus vehicle or that have a negative log2 fold change N/L versus vehicle. (e) Top lysosomal resident proteins found at higher or lower levels in CMA and MA lysosomes. (f) Fold change (over control) in protein levels of TOR protein components found in both CMA and MA lysosomes.

We wondered whether the increased lysosomal activity of senescent cells is accompanied by changes in the proteins undergoing degradation (delivered into lysosomes by CMA or MA), and in constitutive resident lysosomal proteins (both lumen, membrane integral, and membrane‐associated proteins). For this, we isolated lysosomes from control and palbociclib‐induced senescent SK‐MEL‐103 cells using a previously described method based on differential centrifugation and flotation in gradients of discontinuous densities (Cuervo et al., 1997) that allows separation of two fractions enriched in CMA‐ and MA‐engaged lysosomes, respectively. As expected (Koga et al., 2010), CMA lysosomes presented higher levels of the chaperone HSC70 compared to MA lysosomes (Figure 4a). The levels of HSC70 were similarly high in control and senescent CMA lysosomes. However, HSC70 was further reduced in senescent MA lysosomes compared to control ones (Figure 4a), which is in agreement with the higher rates of MA observed in senescent cells, and with the previously reported degradation of luminal lysosomal HSC70 upon fusion with autophagosomes due to transient dissipation of the MA lysosomes luminal pH (Kaushik et al., 2008). Analysis of the lysosomal fractions (CMA and MA) isolated from control and senescent cells revealed comparable total protein levels and specific activity of enzymes such as hexosaminidase (Figure S2A,B), in further support that lysosomes from senescent cells were able to efficiently degrade the increased amount of cargo delivered by MA and CMA to these compartments. Furthermore, we did not find significant differences between the lysosomal fractions isolated from control and senescent cells in lysosomal recovery (percentage of total cellular hexosaminidase activity recovered in the lysosomal fractions), lysosomal purity (enrichment of hexosaminidase activity in the lysosomal fractions relative to total cellular activity) or in the integrity of the lysosomal membrane (percentage of hexosaminidase activity detected outside lysosomes because of breakage of their membranes) (Figure S2C–E). Integrity of the lysosomal fraction (breakage <15%) is an essential requirement to accurately assay CMA and MA, and consequently, only isolations showing hexosaminidase release below those levels were used for these experiments (Figure S2F). Interestingly, comparison of the proteolytic capacity of the luminal content of lysosomes isolated from senescent cells, once corrected per amount of protein, was not higher than in lysosomes isolated from control cells (Figure S2F), suggesting that the increase in MA and CMA flux in senescent cells was not a consequence of faster luminal proteolysis but rather of higher delivery of cargo to lysosomes.

After validating our isolation method, we analyzed the lysosomal proteome (cargo and resident proteins) by mass spectrometry (MS). Of note, MS of lysosomes allows for the detection of resident lysosomal proteins, and it can also detect peptides derived from lysosomal substrates that have been degraded inside the lysosomes during the hours immediately prior to isolation. We treated control and senescent SK‐MEL‐103 cells with N/L for 16 h to block lysosomal proteolysis before lysosomal isolation in order to distinguish lysosomal substrates from resident lysosomal proteins. This allowed us to classify the proteins that increase in the lysosomes upon N/L treatment as lysosomal substrates, and the proteins that do not change or decrease upon N/L as resident lysosomal proteins, as previously described (Schneider et al., 2014) (Figure 4b). We performed three biological replicates on separate days, and each followed an independent purification process. After proteomic analysis by MS, we identified more than 3,400 proteins in the CMA lysosomes, and a similar amount was detected in the MA lysosomes. Principal component analysis (PCA) revealed a consistent clustering of the different biological entities. The first component that accounted for 54% of the differences was the subtype of lysosome, that is, CMA or MA. The second component (11% of variation) separated samples according to the state of cells, that is, non‐senescent (CTRL) or senescent (SEN) (Figure 4c). We conclude that the autophagic pathway (CMA or MA) and the cellular state (non‐senescent or senescent) are associated with a specific protein composition of lysosomes.

### 2.5 Lysosomal resident proteins in senescent cells

**FIGURE 5:**
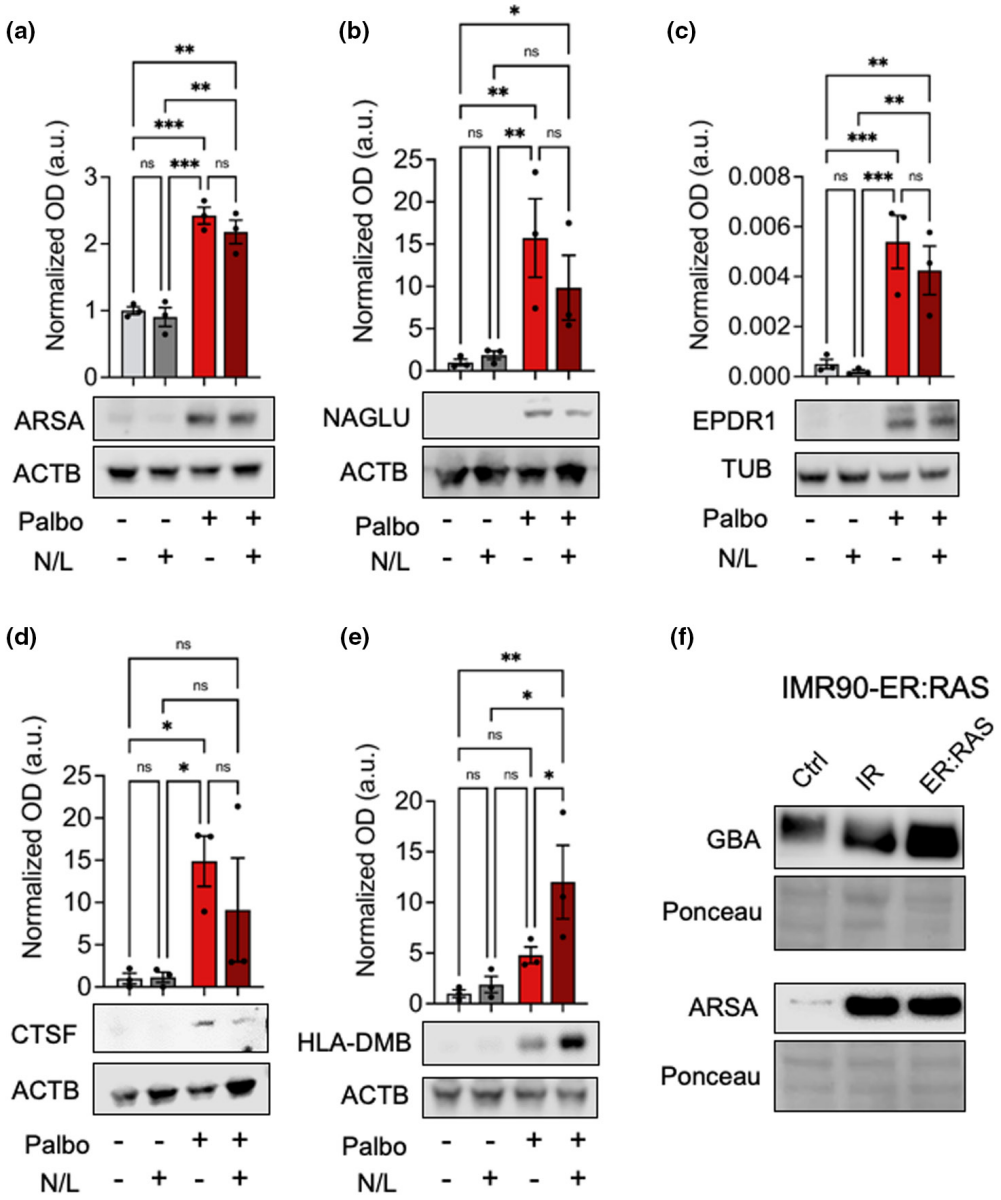
Validation of elevated levels of resident lysosomal proteins in whole‐cell extracts. (a) Immunoblot of ARSA (arylsulfatase A) in total cell homogenates from control or palbociclib‐treated (palbo) SK‐MEL‐103, treated or not with ammonium chloride and leupeptin (N/L) 16 h before cell lysis, as indicated. Quantification of three biological independent replicates (*n* = 3) (top) and representative immunoblot (bottom). ***p < 0.001, **p < 0.01, *p < 0.05, 2‐way ANOVA test. (b) Immunoblot of NAGLU (N‐acetyl‐alpha‐glucosaminidase), same as in panel (a). (c) Immunoblot of EPDR1 (ependymin related 1), same as in panel (a). (d) Immunoblot of CTSF (cathepsin F), same as in panel (a). (e) Immunoblot of HLA‐DMB (beta subunit of MHC‐II DM), same as in panel (a). (f) Immunoblot of IMR90‐ER:RAS from control and senescent cells. Senescence was induced by irradiation (20 Gy, 2 weeks) or by addition of tamoxifen (1 μM, 3 weeks).

Lysosomes from senescent cells presented remarkable changes in the levels of resident proteins (i.e., those that are not degraded and therefore do not accumulate upon N/L treatment). There was a general direct correlation between the changes observed in CMA and MA lysosomes upon senescence (4d,e). For example, we identified 435 resident proteins that were enriched in both CMA and MA lysosomes from senescent cells compared to lysosomes from control cells (Figure 4d,e; Table S1). The top highest enriched resident proteins in each type of senescent lysosomes, CMA or MA, are indicated (Figure 4e). We wondered if these changes in lysosomal resident proteins could be detected by immunoblotting of whole‐cell extracts. We tested the following proteins: ARSA (arylsulfatase A) which in the MS analysis was elevated in both MA and CMA lysosomes; NAGLU (N‐acetyl‐alpha‐glucosaminidase) and EPDR1 (ependymin related 1), elevated in MA lysosomes; and CTSF (cathepsin F) and HLA‐DMB (beta subunit of MHC‐II DM), elevated in CMA lysosomes. All these tested proteins were strongly elevated in senescent SK‐MEL‐103 cells (Figure 5a–e). In agreement with their classification as resident proteins, most of them did not increase upon N/L treatment (Figure 5a–e). We also tested ARSA in human IMR90‐ER:RAS fibroblasts that were induced into senescence by irradiation or by activation of an inducible oncogenic Ras allele, and as in the case of palbociclib‐induced senescence, we observed a robust elevation of ARSA levels upon senescence (Figure 5f).

Analysis of the Gene Ontology terms of the resident proteins in each lysosomal subpopulation of senescent cells identified changes common to both lysosomal groups, such as an increase in mTOR signaling proteins, including multiple components of the RICTOR complex involved in amino acid sensing and activation of mTOR (Figure 4f; Figure S3). This is consistent with previous observations reporting the enhanced association of mTOR with the lysosomes of senescent cells (Narita et al., 2011). Other proteins were markedly reduced in both lysosomal populations in senescent cells, as it was the case of RhoGTPases, known to participate in lysosomal positioning (Figure S3). Amino acid metabolism was also increased in senescent CMA and MA lysosomes (Figure S4). We also identified changes specific for each of the lysosomal subgroups. For example, CMA lysosomes in senescent cells showed a marked increase in enzymes involved in fatty acid metabolism, and in proteins that participate in phagocytosis and in vesicular fusion, whereas cytoskeleton and endocytosis‐related proteins were reduced (Figures S3 and S4A). MA lysosomes from senescent cells displayed increased levels in enzymes involved in metabolism of amino acids and micronutrients, proteins in the vesicular transport group, as well as mitophagy components, whereas we identified a marked decrease in signaling proteins normally present in the surface of control cells lysosomes (Figures S3 and S4B).

### 2.6 Lysosomal protein substrates in senescent cells

**FIGURE 6:**
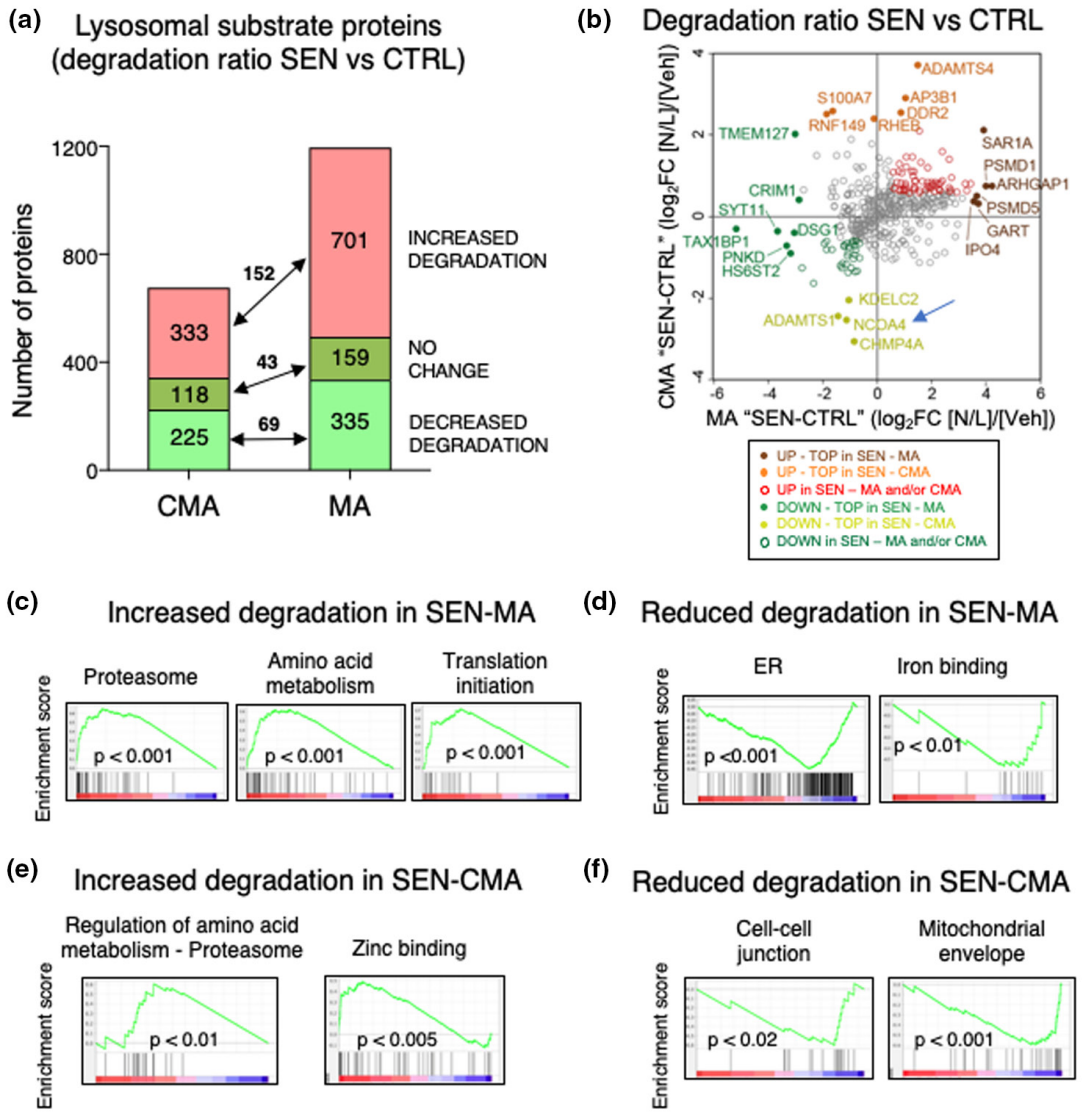
Mass spectrometry analysis of lysosomal substrate proteins. (a) Proteins degraded at higher or lower rates in CMA and MA lysosomes from senescent cells. Substrate proteins were defined as those that accumulate significantly upon N/L treatment, that is, log2 fold change N/L versus vehicle >0.21 (fold >1.1) and *p* value <0.05. Red, substrate proteins that display increase degradation rates in senescence were defined as those either exclusively degraded in senescent cells, or degraded in both, but with a degradation fold change ≥0.6. Dark green, substrate proteins degraded with a similar rate in both conditions (degradation fold change <0.6 and >−0.6). Light green, substrate proteins that display lower degradation in senescence were defined as those exclusively degraded in control, or with a degradation fold change ≤−0.6. (b) Top lysosomal substrate proteins displaying higher or lower rates of lysosomal degradation in CMA and MA lysosomes. (c) GSEA plots of the top c5 GO terms gene sets that were significantly enriched in the proteins displaying increased degradation by MA lysosomes in senescence. (d) Same as in (c) but showing gene sets that were significantly enriched in the proteins displaying lower degradation by MA lysosomes in senescent cells. (e) Same as in (c) but showing gene sets that were significantly enriched in the proteins displaying increased degradation by CMA lysosomes in senescence. (f) Same as in (c) but showing gene sets that were significantly enriched in the proteins displaying decreased degradation in CMA lysosomes in senescent cells.

We next focused on lysosomal protein substrates (i.e., those that are degraded and therefore accumulate upon N/L treatment). We refer to the ratio between N/L and vehicle conditions as degradation ratio (which encompasses lysosomal delivery and proteolysis inside lysosomes) and for each substrate protein we generated a degradation ratio in senescent (SEN) and control (CTRL) cells. Finally, these two degradation ratios were divided (SEN vs. CTRL) to obtain the “degradation fold change” (Table S2). For CMA lysosomes, we found 333 proteins that displayed enhanced lysosomal degradation in senescent cells and 225 proteins whose lysosomal degradation was reduced (Figure 6a). For MA lysosomes, we found 701 proteins with increased lysosomal degradation rates in senescent cells and 335 proteins that displayed lower degradation rates in these lysosomes (Figure 6a; Table S2). Since we did not find differences in the proteolytic capacity of the luminal proteases per se once the lysosomal membrane was disrupted (Figure S2F), we interpret that the differences in degradation rate of specific proteins mostly reflect their rate of delivery to lysosomes. The top proteins with increased or decreased degradation in each type of lysosomes in senescent cells are shown in Figure 6b. Besides changes in degradation rate, we also identified proteins no longer degraded by MA (37) or CMA (83) in senescent cells, as well as proteins degraded through these pathways (165 for MA and 47 for CMA) only in senescent cells but not in control cells (Figure S5). We conclude that senescence entails extensive changes in lysosomal substrates, which are likely consequence of changes in their delivery rates.

To understand the consequences of these quantitative and qualitative changes in autophagy‐mediated degradation in senescence, we first focused on the lysosomal substrates for MA. We analyzed our dataset by gene set enrichment analysis (GSEA) using the c5 Gene Ontology (GO) terms database. Interestingly, we found a significant enrichment in proteasomal machinery gene sets (Figure 6c; Table S2). In relation to this, MA is known to contribute to proteasome degradation under stress conditions such as starvation (Cohen‐Kaplan et al., 2016; Cuervo et al., 1995). We also found an increased degradation rate via MA for translation initiation factors, particularly for several subunits of the eukaryotic translation initiation factor 3 (eIF3) and components of amino acid metabolism pathways including tRNA synthetases (Figure 5c; Table S2). This suggests that senescent cells have a high turnover of the protein translation and quality control machineries, probably due to aberrant protein accumulation. Among the group of proteins not normally degraded by MA, but that become MA substrates in senescent cells, we found a large number of proteins related to the organization of the actin and microtubule cytoskeletons (Figure S5A), which could be behind the reorganization of the cytoskeleton recently described to occur in senescent cells (Moujaber et al., 2019).

Among the protein categories undergoing less degradation by MA during senescence, we found endoplasmic reticulum (ER) components and iron‐binding proteins (Figure 6d; Figure S5B;Table S2). This suggests that the ER turnover in senescent cells by MA is lower as compared to control cells, which is consistent with the secretory nature of senescent cells. Also, in line with the changes in secretion in senescent cells, we identified a subset of proteins involved in constitutive exocytosis (through Golgi transport vesicles that continuously dock in the plasma membrane) that become MA substrates in senescent cells, whereas a different protein subset involved in regulated exocytosis (mediated by secretory vesicles only released in response to a extracellular signal) usually degraded by MA was spared from degradation in senescent cells (Figure S5A,B). These findings support a possible regulatory role for MA in the secretory function and SASP of senescent cells.

We next studied the senescence‐induced changes in the degradation of protein substrates for CMA. Interestingly, GSEA also showed an enrichment in proteasome components under the category of “Regulation of cellular amino acid metabolism” in the substrates degraded more via CMA in senescence, along with zinc‐binding proteins (Figure 6e; Table S2). Degradation of specific proteasome subunits by CMA has also been described to play a regulatory role in overall proteasome content and activity (Cuervo et al., 1995; Juste et al., 2021; Schneider et al., 2015). We also analyzed gene sets that displayed significantly downregulated degradation by CMA and found an enrichment in cell–cell junction organization components (Figure 6f; Table S2). Lysosomes are known to regulate cell–cell junctions (Nighot & Ma, 2016), and cell senescence has been associated with disruptions in tight and gap junctions (Krouwer et al., 2012; Xie et al., 1992). Our results suggest that alterations in cellular junctions might be controlled by CMA lysosomes. Lastly, we noticed a large pool of mitochondrial proteins that become CMA substrates in senescent cells, whereas another subset of mitochondrial proteins is spared from degradation through this pathway (Figure 6f; Figure S5C,D). Although CMA cannot degrade mitochondria as a whole organelle, recent studies have demonstrated regulatory degradation of nuclear‐encoded mitochondrial proteins by CMA before they reach mitochondria (Schneider et al., 2014). We propose that the observed changes in CMA activity and in the type of proteins degraded through this pathway in senescent cells may contribute in part to the remodeling of the mitochondrial proteome described in these cells (Catherman et al., 2013; Sabbatinelli et al., 2019; Sullivan et al., 2021).

### 2.7 Lysosomal secretion of SASP factors by senescent cells

**FIGURE 7:**
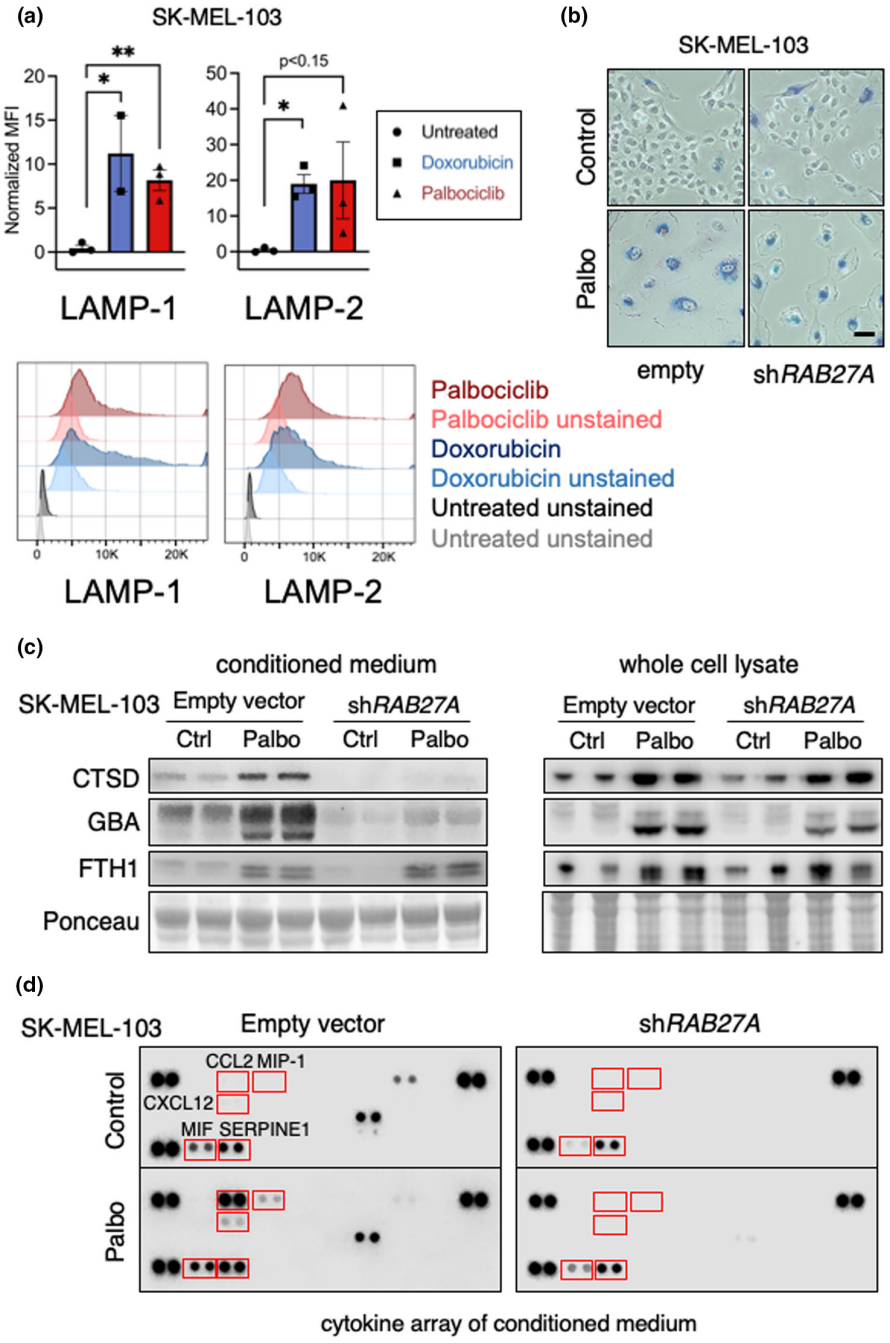
Analysis of lysosomal secretion in senescence. (a) Flow cytometry analysis of LAMP1 and LAMP2 in the surface of alive, non‐permeabilized SK‐MEL‐103 cells, treated or not with 100 nM doxorubicin or 5 μM palbociclib for 1 week. Top, values for three independent biological replicates (*n* = 3), mean ± SD. Due to sample limitation, *n* = 2 in cells treated with doxorubicin and stained for LAMP1. Statistical significance was determined using the unpaired *t*‐test. *p < 0.05, Bottom, example of one flow cytometry assay. (b) SA‐βGal staining pictures of SK‐MEL‐103 cells carrying empty vector or sh*RAB27A*, treated or not with 2 μM palbociclib for 1 week. Scale bar 50 μM. (c) Western blot analysis for CTSD, GBA, and FTH1 in the CM (left) and whole‐cell lysates (right) of SK‐MEL‐103 cells, proliferative or senescent (palbociclib) carrying empty vector or sh*RAB27A*. (d) Cytokine profile of the CM from SK‐MEL‐103 cells carrying empty vector or sh*RAB27A*, treated or not with palbociclib.

Considering the high levels of lysosomes in senescent SK‐MEL‐103 cells, we wondered if senescent cells could be able to perform lysosomal secretion (Settembre et al., 2013). Lysosomal secretion results in the exposure of lysosomal membrane proteins at the plasma membrane. We determined the presence of LAMP1 and LAMP2 by FACS in alive, non‐permeabilized, control, and senescent cells. Consistent with lysosomal secretion, senescent cells presented high levels of LAMP1 and LAMP2 at the plasma member (Figure 7a). To study the mechanism of lysosomal exocytosis in senescence, we focused on RAB27A, a small GTPase that plays a key role in non‐canonical vesicle secretion, including lysosomes (Bahadoran et al., 2001; Haddad et al., 2001; Johnson et al., 2013; Wilson et al., 2000) and extracellular vesicles (EV) (Ostrowski et al., 2010; Peinado et al., 2012). Interestingly, our proteomic analysis detected RAB27A as a resident protein in both MA and CMA lysosomes (levels remained unchanged upon N/L treatment; Figure S6A). First, we used a short hairpin RNA targeting *RAB27A* in SK‐MEL‐103 cells and confirmed efficient reduction of RAB27A at the protein level (Figure S6B). We verified that *RAB27A*‐KD cells can efficiently undergo senescence, as evidenced by positive SAβGal staining seven days after palbociclib treatment (Figure 7b). Finally, we checked for the presence of lysosomal proteins in the conditioned medium (CM) of senescent cells, either wild type (WT) or *RAB27A*‐KD. We found that the lysosomal lumen proteins CTSD and GBA were increased in the CM from senescent cells, while they were undetectable in the CM of *RAB27A*‐KD senescent cells (Figure 7c). To corroborate that RAB27A specifically affects non‐canonical exocytosis, we also analyzed the levels of ferritin, which accumulates in senescent cells (Masaldan et al., 2018) and can be secreted via the canonical ER‐Golgi secretory pathway (Ghosh et al., 2004). Accordingly, ferritin heavy chain 1 (FTH1) was increased in the CM of senescent cells, regardless of the presence or absence of sh*RAB27A* (Figure 7c). As an additional control, the levels of CTSD, GBA, and FTH1 in whole‐cell lysates were similar in WT and *RAB27A*‐KD senescent cells, and higher than in WT non‐senescent cells (Figure 7c). These findings were replicated using a pool of siRNAs targeting multiple sequences of *RAB27A* (Figure S6C).

However, we were not able to detect secreted lysosomal proteins in the CM of senescent IMR90‐ER:RAS (see Section 3). This leaves open the possibility that RAB27A‐dependent lysosomal secretion could be a cell type‐dependent feature of senescent cells, and prompts the need for future studies to evaluate the relative contribution of different secretory mechanisms to the SASP.

To further examine the contribution of RAB27A‐regulated secretion to the SASP, we analyzed the CMs from the previous experiment in an array with immobilized antibodies for 36 cytokines and chemokines. Five proteins present in the CM from WT senescent cells were markedly reduced in the CM from *RAB27A*‐KD cells, namely CCL2, CCL3/4, CXCL12, MIF, and SERPINE1, which are well‐known SASP components (Figure 7d,e; Figure S6D). We wondered whether RAB27A inhibition could affect the mRNA levels of CCL2 and SERPINE1 in senescent cells. However, the mRNA levels of these SASP factors were highly elevated in senescent cells regardless of the inhibition or not of *RAB27A* (Figure S6E). The translation of many cytokines in senescent cells depends on the characteristic high levels of mTORC1 and its downstream effector phospho‐4EBP (Herranz et al., 2015; Laberge et al., 2015). Again, knockdown of *RAB27A* mRNA with siRNAs did not affect the high levels of phospho‐4EBP1 in senescent cells (Figure S6F). Overall, our results indicate that the secretion of some SASP components in senescent melanoma cells occurs, at least in part, via secretory lysosomes in a RAB27A‐dependent manner.

### 2.8 Plasma markers of aging present in the lysosome of senescent cells

It has been recently reported that 25 SASP proteins shared by several types of senescence (Basisty et al., 2020) are also present in a catalogue of human plasma proteins significantly upregulated with aging (Tanaka et al., 2018). We wondered if these 25 “SASP‐and‐aging” proteins could result from lysosomal exocytosis by senescent cells. We generated a ranked list of all the proteins enriched in senescent lysosomes compared to control cells, without taking into account whether the proteins are resident or substrates (Table S3). Interestingly, among the 25 “SASP‐and‐aging” markers previously defined (Basisty et al., 2020), 9 proteins (36%) were enriched in the lysosomes of senescent cells (CMA or MA lysosomes or both) (Table 1). Most of these 9 proteins are typical resident lysosomal proteins including four proteases, namely cathepsins CTSB, CTSD, CTSZ, and metalloproteinase MMP2 (Table 1). These observations suggest that some serum biomarkers of aging may reflect the process of lysosomal secretion by senescent cells.

**TABLE 1:** Lysosomal proteins upregulated in senescent cells in our study that are age‐upregulated serum SASP factors (Basisty et al., 2020)

Protein name	Gene	Fold UP in SEN MA‐lys	Fold UP in SEN CMA‐lys
Cathepsin Z	CTSZ	8.04	3.40
Cathepsin B	CTSB	5.33	4.71
Cathepsin D	CTSD	5.05	5.08
Gelsolin	GSN	4.15	5.79
Fibronectin	FN1	1.89	Not present
Metalloproteinase inhibitor 2	TIMP2	1.77	1.63
Protein deglycase DJ‐1	PARK7	1.62	1.34
Matrix metalloproteinase 2	MMP2	Not present	2.38
Coactosin‐like protein	COTL1	Not present	1.23

## 3 DISCUSSION

One of the main features of senescent cells is their enlarged lysosomal compartment. Here, we have confirmed the previously described increase in MA in senescent cells and identified differences in the kinetics of activation of *in bulk* and selective MA during the establishment of senescence, with *in bulk* MA preceding selective (p62‐ and NBR1‐dependent) MA. In addition, we report for the first time, increased activity of CMA in senescent cells, coincident in time with the switch toward more selective MA in these cells. We demonstrate that the increase in CMA during senescence may be a general phenomenon, since we could detect it in the 5 different cell types tested, including primary human and mouse fibroblasts, as well as immortalized and cancer cells, and in response to different pro‐senescence stimuli. The upregulation of both autophagic pathways, MA and CMA, leads to quantitative and qualitative changes in protein degradation that likely contribute to remodeling of the senescent cell proteome.

Our studies support that the expansion of the lysosomal compartment observed in senescent cells is largely due to de novo lysosome biogenesis, as we found a clear increase in the transcription of TFEB, a key upstream transcriptional regulator of both lysosomal biogenesis and MA, upon induction of senescence. Interestingly, although CMA is not under the regulation of this transcription factor, activation of this type of selective autophagy during senescence is also transcriptionally regulated, as the increase in LAMP2A, the limiting protein in CMA, was noted both at the mRNA and protein levels. Lysosomes not only increase in number in senescent cells, but they also display changes in their protein composition (differences in abundance of lysosomal resident proteins—defined as all those proteins detected in lysosomes that do not undergo degradation, and that include lysosome membrane‐associated proteins, integral membrane proteins, and lysosomal lumen resident enzymes). Differences in the enzymatic content of lysosomes may occur to accommodate the observed changes in the type of cargo delivered to these organelles for degradation in senescent cells. For example, higher content of lipases in lysosomes has been observed upon exposure to high lipid content diets (O'Rourke & Ruvkun, 2013). Although future studies are required to elucidate the specific consequences of some of the changes in the composition of proteins associated with the membrane of lysosomes in senescent cells, it is likely that the observed changes in cytoskeleton‐related proteins and other proteins commonly involved in vesicular trafficking and fusion events in pathways such as exocytosis, endocytosis, and phagocytosis may determine differences in the cellular position of these organelles and in their ability to fuse with other vesicular compartments and with the plasma membrane. Of particular interest among this group of proteins associated with the lysosomal membrane, are those involved in intracellular signaling. The role of lysosomes as platforms for signaling is now well accepted (Lawrence & Zoncu, 2019), but to the best of our understanding, this is the first report of pronounced changes in these lysosome‐signaling pathways in senescent cells. On this respect, we found higher association of several mTOR components to lysosomes in senescent cells, consistent with previous observations reporting an association between mTOR and autolysosomes in senescence, which facilitates protein synthesis (Narita et al., 2011). In contrast to this increase in mTOR components, our proteomic analysis revealed significant reduction in other major pathways that signal from the lysosome such as phospholipase D, Ras, or Hippo pathways (Figure S3B). Future studies on the functional consequences of these changes could provide novel insights on the contribution of lysosomal signaling to the senescent phenotype.

The activation of MA has been reported in replicative senescence and also during oncogene‐induced senescence, where it plays a key role in SASP induction (Gamerdinger et al., 2009; Narita et al., 2011; Young et al., 2009). In the senescence paradigm used in this work, we found that although activation of MA occurs early after exposure to the pro‐senescent agent, there are changes in the level of MA and the type of MA as the senescent phenotype is being stablished. Thus, the two MA receptors analyzed in this work, p62 and NBR1, are not involved in the initial peak of MA activation which can be attributed for the most part to “*in bulk*” MA. However, we noted degradation of these key mediators of selective MA as the senescent phenotype is established, which along with the changes in lysosomal cargo detected in senescent cells, supports a switch toward selective degradation. Analysis of the cellular functions of those proteins with different MA degradation in senescent cells when compared to control suggests that MA actively contributes to quality control of the high load of de novo translated secretory proteins, maintenance of the ER and of the protein translation machinery. Thus, we found augmented degradation of translation initiation factors such as eIF3, and of tRNA synthetases. In this regard, studies in yeast have shown that eIF3 can assemble into a large supercomplex, the translatome, which contains elongation factors, tRNA synthetases, 40S and 60S ribosomal proteins, chaperones, and the proteasome (Sha et al., 2009). Further studies are needed to elucidate the link between protein synthesis and degradation in senescence. MA in senescent cells may also mediate cellular structural changes, such as remodeling of the cytoskeleton and turnover of the proteasome. Of note, impaired proteasome function has been tightly correlated to senescence and aging (Chondrogianni et al., 2003; Chondrogianni & Gonos, 2004, 2005; Stratford et al., 2006).

In contrast to MA, the process of CMA has remained unexplored in senescent cells. We have identified that CMA is upregulated in response to a variety of senescence‐inducing agents independently of the cell type. The subpopulation of lysosomes preferentially dedicated to CMA (about 30% of cellular lysosomes under basal conditions) also undergoes qualitative and quantitative changes in their proteome upon senescence thus suggesting that both MA and CMA lysosomes reprogram upon senescence induction. The subset of the cellular proteome undergoing degradation via CMA also changes during senescence. These changes in CMA cargo in senescence support a possible contribution of CMA to the remodeling of the mitochondrial proteome and subsequent functional changes in this organelle, by promoting degradation of part of its proteome while preserving other part. Similarly, reduced CMA degradation of tight junction components in senescent cells may lead to the disruptions in tight and gap junctions observed in these cells (Krouwer et al., 2012; Xie et al., 1992) by altering the normal stoichiometry of their components.

Future studies are needed to elucidate the cellular consequences and contribution to senescence of the observed increase in autophagic degradation of specific proteins in senescent cells. Expanding our findings on lysosomal degradation with senescence‐induced changes at the transcriptional and/or translational level for each specific protein would help to conclusively address whether changes in degradation are part of overall increased turnover of proteins under senescence conditions, or if the increased degradation is a means to adjust cellular levels of specific proteins. Based on the substrates with enhanced degradation revealed by the proteome analysis, we favor more a regulatory function for the changes in autophagy during senescence. For example, increased lysosomal degradation of specific proteasome subunits, such as PSMD5, may reduce its cellular levels and thus prevent the previously described ameliorating effect of PSMD5 on replicative senescence (Lu et al., 2014). Coordinated changes during senescence in protein degradation and synthesis may allow senescent cells to transition through the different stages of senescence.

Senescent cells can strongly affect the microenvironment and promote tissue remodeling through the secretion of pro‐inflammatory cytokines, chemokines, growth factors, and proteases, known as the senescence‐associated secretory phenotype or SASP (Coppé et al., 2008). Some cell types have the capacity to perform lysosomal exocytosis as part of their normal physiology, in the case of osteoclasts, melanocytes, endothelial cells, and cells from the hematopoietic lineage, including lymphocytes, neutrophils, mast cells, and macrophages (Blott & Griffiths, 2002; Mostov & Werb, 1997; Settembre et al., 2013; Stinchcombe et al., 2004). We observed that a significant number of previously reported SASP proteins (Basisty et al., 2020), which have also been described as human aging biomarkers in plasma (Tanaka et al., 2018), are enriched in the lysosomes of senescent cells. In fact, most of these proteins are lysosomal, such as the aspartyl protease, CTSD. Our finding that secretion of these lysosomal components in melanoma cells is dependent on RAB27A, a protein that we found associated with both lysosomal populations analyzed in this study, highlights this GTPase as a potential future target to modulate SASP in aging. Of note, we also observed that some cytokine characteristic of the SASP are also secreted in a RAB27A‐dependent manner. In particular, a cytokine array screening identified that the secretion of CCL2, CCL3/4, CXCL12, MIF, and SERPINE1 from senescent cells is dependent on RAB27A. Given the fact that RAB27A participates in various unconventional secretory processes, including lysosomal secretion and extracellular vesicles (EV) secretion, it remains open if these cytokines are secreted exclusively via lysosomes. It is important to mention that we were not able to detect secreted lysosomal proteins in the CM of senescent IMR90‐ER:RAS. This leaves open the possibility that RAB27A‐dependent lysosomal secretion could be a cell type‐dependent feature of senescent cells, and prompts the need for future studies to evaluate the relative contribution of different secretory mechanisms to the SASP.

Overall, we have observed that senescent cells upregulate CMA together with MA, and that the protein content in the lysosomes changes significantly upon senescence induction. We have also shown that lysosomal secretion may contribute to the SASP. Finally, our comprehensive catalogue of lysosomal proteins can be used to further elucidate the interplay between autophagy and senescence, and to identify novel in vivo biomarkers secreted by senescent cells.

## 4 METHODS

### 4.1 Cell lines and media

Cell lines SK‐MEL‐103 (human melanoma), Huh7 (human hepatocarcinoma), U2OS (human primary osteogenic sarcoma), NIH 3T3 (mouse fibroblasts), Neuro2a cells (mouse neuroblastoma), and SAOS‐2 (human primary osteogenic sarcoma) were obtained from ATCC. Human fibroblasts (IMR90 PDL 20) were from Coriell Repository, and mouse embryonic fibroblasts from wild‐type mice were prepared in our laboratory as described before (Schneider et al., 2014). Human fibroblasts IMR90 ER:RAS were kindly provided by Daniel Munoz (University of Cambridge). To induce *RAB27A*‐KD, the siRNA produced by siTOOLs was used. Cells were transfected using lipofectamine RNAiMAX Transfection Reagent (13778030, Life Technologies). After 5 days, the cells and the conditioned media (CM) were harvested for further analyses. To produce the CM, plated cells were washed 3 times with FBS‐free medium for 10 min at 37°C each. The cells were then incubated in 0.1% FBS‐free medium (12 ml per 15 cm plate) for 24 h at 37°C. CM was collected and centrifuged at 1200 g for 5 min and then concentrated (1:100 fold) using 3.5 kDa Amicon centrifugal filter units (UFC900324, Millipore). All cell lines were maintained in DMEM supplemented with 10% fetal bovine serum or newborn calf serum and penicillin–streptomycin (all from Gibco), and incubated in 20% O_2_ and 5% CO_2_ at 37°C (the only exception were IMR90 ER:RAS cells that were maintained in a hypoxia incubator at 3% O_2_). Cells were routinely tested for mycoplasma contamination using the mycoplasma tissue culture NI (MTC‐NI) Rapid Detection System (Gen‐Probe). For senescence induction, cells were supplemented with media containing palbociclib (1 μM) (PD033299, Pfizer Inc.) or doxorubicin (100 nM) (Sigma, #D1515), as indicated in the figure legends. In the case of NIH3T3 cells, we induced senescence by addition to the culture media of palbociclib (1 μM), etoposide (50 μM) (Sigma, # E1383), paraquat (50 μM) (Sigma, #P856177), and BIBR1532 (1 μM) (Tocris, Cat. No. 2981) for 72 h. For oncogene‐induced senescence, IMR90 ER:RAS cells were treated with 4‐OH tamoxifen (1 μM) (Sigma, #H7904) for 3 weeks. For irradiation‐induced senescence, IMR90 ER:RAS cells were irradiated at 20 Gy and cells were analyzed after 2 weeks. For inhibition of lysosomal function, cells were treated with NH_4_Cl (Sigma, # 254134) at 100 mM and leupeptin hemisulfate (Fisher BioReagents, #BP2662) at 100 μM (N/L) for 16 h, as indicated in the figure legends. For shRNA‐mediated knockdown of *RAB27A*, we transfected HEK293T (5 × 10^6^) cells with empty lentiviral pLKO.1vector (empty vector) or encoding shRNA against human *RAB27A* (TRCN0000005295: 5′‐CCAGTGTACTTTACCAATATA‐3′) (purchased from Sigma), and packaging vectors using Fugene HD (Roche). Viral supernatants were collected 36 h after transfection and were used to infect WT SK‐MEL‐103, previously plated at a density of 8 × 10^5^ cells per 10 cm plates. Prior to infection, polybrene was added to the viral supernatants at a concentration of 8 mg/ml. Stable transformants were selected by their resistance to puromycin. Treatments with senescence‐inducing agents were performed after confirmation of stable knockdown. For siRNA‐mediated knockdown of *RAB27A*, we used the commercial pool of siRNAs commercialized by siTOOLs against *RAB27A* (as controls we used non‐targeting siRNAs from the siTOOLs, siNT). Cells were transfected using lipofectamine RNAiMAX Transfection Reagent (13778030, Life Technologies). After 5 days, the cells and the conditioned media were harvested for further analysis. To produce the conditioned medium (CM), plated cells were washed 3 times with medium without serum for 10 min at 37°C each. The cells were then incubated in 0.1% FBS medium (12 ml per 15 cm plate) for 24 h at 37°C. CM was collected and centrifuged at 1200 g for 5 min, and then concentrated (1:100 fold) using 3.5 kDa Amicon centrifugal filter units (UFC900324, Millipore).

### 4.2 Intracellular protein degradation assay

Measure of intracellular degradation was performed in SK‐MEL‐103 cells by metabolic labeling with 2 μCi/ml ^3^H‐leucine (PerkinElmer) for 48 h at 37°C and pulse‐chase experiments as described previously (Auteri et al., 1983). After labeling, cells were extensively washed and maintained in medium with an excess of unlabeled leucine. Aliquots of the medium were taken immediately after washing and at different times for 24 h and were precipitated in trichloroacetic acid. Proteolysis was measured as the amount of acid‐precipitable radioactivity transformed in acid‐soluble radioactivity at each time point. Lysosomal‐dependent degradation was inhibited using 100 mM NH_4_Cl and 100 μM leupeptin (N/L).

### 4.3 Autophagy reporter assays

SK‐MEL‐103 cells were transduced with a lentivirus carrying the photo‐switchable KFERQ‐PS‐Dendra2 CMA reporter, a brighter modified version of the original reporters (Caballero et al., 2018; Dong et al., 2020, 2021; Juste et al., 2021; Koga et al., 2011) to measure CMA, or with a lentivirus carrying a tandem fluorescent LC3 construct (mCherry‐GFP‐LC3; Kimura et al., 2007) to measure MA. After transduction with either of the viruses, cells were selected by resistance to puromycin for 2 weeks and then used for the experiments in puromycin‐free media. Instead of selection of multiple single clones, we have found higher reproducibility when working with the pool of transduced cells to account for the variety of insertion sites of the lentivirus. Transduced cells were treated with 1 μM palbociclib for 1 week for senescence induction. Cells transduced with the KFERQ‐PS‐Dendra2 reporter were photoswitched with a 405‐nm light‐emitting diode (Norlux) for 4 min (in 96‐well plates) or 7 min (in coverslips) with the intensity of 3.5 mA (current constant) 16 h before imaging (in the standard 7 days palboclib treatment) or 72 h before imaging (in the studies in which senescence was induced for 3 days). For imaging, cells were plated in 96‐well plates with glass‐bottom or in glass coverslips (depending on the cell type) and fixed with 4% paraformaldehyde. To acquire images from the 96‐well plates, we used a high‐content microscope (Operetta System, Perkin Elmer) and quantification was performed with the manufacturer's software in a minimum of 800 cells (approx. 9 fields). In all cases, focal plane thickness was set at 0.17 μm and sections with maximal nucleus diameter were selected for quantification. Values are presented as number of puncta per cell section that in our acquisition conditions represents 10%–20% of the total puncta per cell. The number of fluorescent puncta by cell was quantified using Image J (NIH) in individual single planar images after thresholding.

### 4.4 Macroautophagy flux

Macroautophagic flux was measured in protein lysates using immunoblot for LC3‐II, p62, or NBR1 in cells treated or not with lysosomal protease inhibitors (10 mM ammonium chloride and 100 μM leupeptin). Flux was calculated as the increase in levels of LC3‐II in protease inhibitors‐treated cells relative to untreated cells.

### 4.5 Cytokine array

Cytokine levels in conditioned medium (CM) were analyzed using the Proteome Profiler Human Cytokine Array (R&D Systems; #ARY005B), following the manufacturer's instructions. Pixel density was determined using the ImageStudio Software.

### 4.6 Lysosomal isolation

Lysosomes were isolated from SK‐MEL‐103 cells after disruption of the plasma membrane by nitrogen cavitation and sequential centrifugation of the light mitochondrial‐lysosomal fraction in a discontinuous metrizamide density gradient, as previously described (Cuervo et al., 1997; Storrie & Amadden, 1990). Briefly, cells were washed two times with PBS and harvested by scrapping with a rubber policeman. After washing the pellet with 0.25 M sucrose, cells were subjected to nitrogen cavitation for 7 min at 36 psi and then homogenized with 8 strokes of a teflon pestle in a Dounce tissue homogenizer (Wheaton) on ice. Broken cell homogenates were centrifuged at 2500× *g* for 15 min at 4°C, and the post‐nuclear pellet was discarded. Then, supernatants were centrifuged at 100,000× *g* for 1 h at 4°C to pellet the light mitochondria and lysosomes fraction. The supernatant was retained and centrifuged at 100,000× *g* for 1 h at 4°C to collect the cytosolic fraction from the resulting supernatant. The mitochondrial‐lysosomal fraction was washed once in 0.25 M sucrose and resuspended in 57% metrizamide (final concentration), and a discontinuous gradient of metrizamide was constructed (layers from the bottom to top were 57%, 32.8%, 26.3%, and 19.8%) and then centrifuged for 1 h at 141,000× *g*. Lysosomes enriched for CMA activity were collected from the top layer (P1), and the mixed population of lysosomes present in the 26.3%/19.8% interface (P2) was collected separately. Both P1 and P2 lysosomal populations were separately resuspended in 0.25 M sucrose and washed by centrifugation at 37,000× *g* for 15 min at 4°C. Then, the P2 fraction was resuspended in 0.25 M sucrose and centrifuged at 100,000× *g* for 5 min at 4°C. The supernatant was used to resuspend the P1 pellet, obtaining the final sample of CMA‐enriched lysosomes. The P2 pellet was resuspended in 0.25 M sucrose to obtain the final sample of MA lysosomes. Lysosomal membrane integrity was verified by comparative analysis of β‐Hexosaminidase activity for intact lysosomes or 1% Triton X‐100‐lysed lysosomes. Β‐Hexosaminidase activity was measured using the membrane impermeant fluorogenic substrate 4‐methylumbelliferyl‐N‐acetyl‐β‐glucosaminide dehydrate (Sigma, #M2133) as previously described (Rodríguez et al., 1997). Only lysosomes with an integrity of >85% were used in subsequent assays.

### 4.7 Analysis of mRNA levels

Total RNA was isolated by acid guanidinium thiocyanate‐phenol‐chloroform extraction. Up to 4 μg of total RNA was reverse transcribed into cDNA using iScriptTM Advanced cDNA Synthesis Kit for RT‐qPCR (BioRad #172‐5038). For qRT‐PCR, each cDNA sample was assayed in triplicate reactions in optical‐grade, 98‐ or 386‐well plates (Applied Biosystems). Each reaction contained 6 μl of SybrGreen (2x) (Applied Biosystems), 2 μl of cDNA template, 3.4 μl of DNAse‐free water, and 0.6 μl of primers (10 μM final concentration). All PCR runs were performed on a QuantStudioTM 6 Flex Real‐Time PCR System using QuantStudioTM 6 and 7 Flex Real‐Time PCR software v1.0 (Applied Biosystems). Reaction conditions were as follows: 10 min at 95°C followed by 40 cycles of 15 s at 95°C, 1 min at 60°C and 1 min at 62°C. The housekeeping genes *ACTB*, *18S rRNA*, and *B2M* were used for input normalization, as indicated.

PCR primers:

*ACTB* Forward: 5′‐CATGTACGTTGCTATCCAGGC‐3′
*ACTB* Reverse: 5′‐CTCCTTAATGTCACGCACGAT‐3′
*18S rRNA* Forward: 5′‐GTAACCCGTTGAACCCCATT‐3′
*18S rRNA* Reverse: 5′‐CCATCCAATCGGTAGTAGCG‐3′
*B2M* Forward: 5′‐CCCAAGATAGTTAAGTGGGATCGA‐3′
*B2M* Reverse: 5′‐CCAAATGCGGCATCTTCAA‐3′
*LAMP1* Forward: 5′‐TTTGGCTCTGTGGAGGAGTG‐3′
*LAMP1* Reverse: 5′‐ACGTTTGCCAGAAAGTGTGC‐3′
*LAMP2A* Forward: 5′‐TGACGACAACTTCCTTGTGC‐3′
*LAMP2A* Reverse: 5′‐AGCATGATGGTGCTTGAGAC‐3′
*LAMP2B* Forward: 5′‐TGCTGGTCTTTCAGGCTTGATT‐3′
*LAMP2B* Reverse: 5′‐TTGCATGTTGGAACTTGTACTTGC‐3′
*TFEB* (transcript variant 2) Forward: 5′‐TTCCAACAAGGGAA GGTGAC‐3′
*TFEB* (transcript variant 2) Reverse: 5′‐CTGCATGCGCAACC CTAT‐3′
*CCL2 (MCP1*) Forward: 5′‐AGAATCACCAGCAGCAAGTGTCC‐3′
*CCL2 (MCP1*) Reverse: 5′‐TCCTGAACCCACTTCTGCTTGG‐3′
*SERPINE1 (PAI1)* Forward: 5′‐CTCATCAGCCACTGGAAAGGCA‐3′
*SERPINE1 (PAI1)* Reverse: 5′‐GACTCGTGAAGTCAGCCTGAA AC‐3′
*ACTB* Forward: 5′‐CATGTACGTTGCTATCCAGGC‐3′
*ACTB* Reverse: 5′‐CTCCTTAATGTCACGCACGAT‐3′
*GAPDH* Forward: 5′‐GGATTTGGTCGTATTGGG‐3′
*GAPDH* Reverse: 5′‐GGAAGATGGTGATGGGATT‐3′

### 4.8 Western blot

Total cell lysates and subcellular fractions in the isolation experiments were prepared in 0.25 M sucrose containing protease and phosphatase inhibitors (Sigma‐Aldrich, #P5726, #P0044). For direct immunoblot analyses of whole‐cell extracts, cells were lysed in lysis buffer (50 nM Tris–HCl pH 8, 1 mM EDTA, 150 mM NaCl, 1% NP40, 0.5% Triton X‐100, 1% SDS with freshly added protease inhibitors; Roche #11873580001), and 15 μg of cell lysate were loaded per lane and hybridized overnight at 4°C. Samples were subjected to SDS‐PAGE, wet‐transferred to nitrocellulose membranes, and blocked with 5% low‐fat milk in TTBS. Blots were incubated with the following primary antibodies mouse anti‐LAMP1 (1:3000, Hybridoma Bank, #H4A3), rabbit anti‐LAMP2A (1:1000, Abcam, #ab18528), mouse anti‐LAMP2 (1:3000, Hybridoma Bank, #H4B4), rabbit anti‐LC3B (1:1000, Cell Signaling, #3868), mouse IgM anti‐HSC70 13D3 (1:1000, Novus Biologicals, #nb120‐2788), goat anti‐CTSD C20 (1:1000, Santa Cruz, #sc‐6486), rabbit anti‐GBA (1:1000, Sigma, #G4171), rabbit anti‐VPS4 (1:1000, Sigma, #sab4200025), rabbit anti‐p62 (1:500 Abcam, #ab155686), mouse anti‐NBR1 (1:500, Abnova, #h00004077‐b01p), mouse anti‐HMGB1 (1:5000, Abcam, #ab18256), rabbit anti‐EPDR1 (1:500, Abcam, #ab197932), rabbit anti‐ARSA (1:500, Abcam, #ab174844), rabbit anti‐HLA‐DMB (1:500, Abcam, #ab131273), rabbit anti‐NAGLU (1:500, Abcam, #ab214671), rabbit anti‐CTSF (1:500, Abcam, #ab200650), rabbit anti‐pospho‐4E‐BP1(Thr37/46) (1:500, Cell Signaling, #2855S), mouse anti‐ACTINB (1:5000, Merck, #A5441), and mouse anti‐G‐Tubulin (1:5000, Merck, #T6557). Proteins were visualized either by using peroxidase‐conjugated secondary antibodies and chemiluminescent reagent (PerkinElmer, #NEL104001EA) in a LAS‐3000 Imaging System (Fujifilm) or using secondary fluorescent reagents (goat anti‐mouse or anti‐rabbit IRDye 680RD and 800CW, LI‐COR). Signal from each antibody was normalized using housekeeping proteins (ACTB or TUBG1) as indicated.

### 4.9 Mass spectrometry

Lysosome samples were solubilized to a final concentration of 5% SDS, 50 mM TEAB pH 7.55. Protein concentration was determined using the micro‐BCA method with BSA as standard. Then, 25 μg of each sample was digested by means of the Protifi™ S‐Trap™ Mini Spin Column Digestion Protocol. Briefly, proteins were reduced and alkylated (15 mM TCEP, 25 mM CAA) 1 h at 45°C in the dark. SDS was removed from samples using 90% methanol in 100 mM TEAB, and proteins were digested with 50 μl of trypsin in 50 mM TEAB pH 7.55 (Trypzean, Sigma, protein: enzyme ratio 1:25, 16 h at 37°C). Resulting peptides were eluted from S‐Trap columns, speed‐vac dried, re‐dissolved in 25 μl of 0.5% formic acid, and analyzed without further desalting.

LC–MS/MS was done by coupling an UltiMate 3000 *RSLCnano* LC system to a Q Exactive HF mass spectrometer (ThermoFisher Scientific). 2 μl of peptides was loaded into a trap column (Acclaim™ PepMap™ 100 C18 LC Columns 5 μm, 20 mm length) for 3 min at a flow rate of 10 μl/min in 0.1% FA. Then, peptides were transferred to an EASY‐Spray PepMap RSLC C18 column (Thermo) (2 μm, 75 μm × 50 cm) operated at 45°C and separated using a 60 min effective gradient (buffer A: 0.1% FA; buffer B: 100% ACN, 0.1% FA) at a flow rate of 250 nl/min. The gradient used was, from 4% to 6% of buffer B in 2 min, from 6% to 33% B in 58 min, plus 10 additional min at 98% B. Peptides were sprayed at 1.8 kV into the mass spectrometer via the EASY‐Spray source, and the capillary temperature was set to 300°C. The mass spectrometer was operated in a data‐dependent mode, with an automatic switch between MS and MS/MS scans using a top 15 method (intensity threshold ≥6.7e4, dynamic exclusion of 25 s, and excluding charges +1 and >+6). MS spectra were acquired from 350 to 1400 m/z with a resolution of 60,000 FWHM (200 m/z). Ion peptides were isolated using a 2.0 Th window and fragmented using higher‐energy collisional dissociation (HCD) with a normalized collision energy of 27. MS/MS spectra resolution was set to 15,000 (200 m/z). The ion target values were 3e6 for MS (maximum IT of 25 ms) and 1e5 for MS/MS (maximum IT of 15 ms).

Data were processed with MaxQuant (v 1.6.0.16) using the standard settings against a human protein database (UniProtKB/TrEMBL, 20,373 sequences) supplemented with contaminants. Carbamidomethylation of cysteines was set as a fixed modification, and oxidation of methionines and protein N‐term as variable modifications. Minimal peptide length was set to 7 amino acids, and a maximum of 2 tryptic missed‐cleavages were allowed. Results were filtered at 0.01 FDR. For label‐free quantification match between runs, option was enabled. Then, the “proteinGroups.txt” file was loaded in Prostar (v1.14) (Wieczorek et al., 2017) using the intensity values for further statistical analysis. Briefly, proteins with less than 4 valid values in at least one experimental condition were filtered out. Then, global normalization of log2‐transformed intensities across samples was performed using the LOESS function. Missing values were imputed using the algorithms SLSA (for partially observed values) and DetQuantile (for values missing on an entire condition). Differential analysis was done using the empirical Bayes statistics limma. Proteins with a *p* value <0.05 and a log2 ratio >1 or <−1 were defined as regulated. FDR was estimated to be <5% by Benjamini–Hochberg. GSEA was performed using the GSEA software v2.0.6 from the Broad Institute. Differentially expressed proteins were ranked according to their log2 ratio and used as input for the enrichment analysis. All fields were set to default, and only gene sets significantly enriched at a FDR q‐values <0.25 were considered. GO analysis was performed using the STRING functional protein association networks (https://string‐db.org/) and Reactome (https://reactome.org/).

The proteomic data are openly available in ProteomeXchange at [https://www.ebi.ac.uk/pride/], with identifier PXD022492.

### 4.10 Flow cytometry analysis

For flow cytometry analysis, SK‐MEL‐103 cells were first treated with 100 nM doxorubicin or 5 μM palbociclib for 1 week. Then, both control and senescent cells were digested into single cells by trypsinization (0.25% trypsin‐EDTA (Invitrogen) at 37°C for 5 min). Cells were not permeabilized. Cell viability was assessed using Live/Dead Fixable Yellow dye (Invitrogen, #L34967) following manufacturer indications, to exclude dead cells from the analysis. Cells were stained with primary antibodies against human LAMP1 (Hybridoma Bank, #H4A3) and LAMP2 (Hybridoma Bank, #H4B4) diluted in FACS buffer (PBS containing 2 mM EDTA 0.5% BSA) for 30 min at 4°C in the dark. A fluorophore‐conjugated secondary antibody (Invitrogen, #A11029) was used. After staining, cells were washed and resuspended in FACS buffer. Cell suspensions were run on a Galios Beckman Coulter flow cytometer (BD Biosciences). Autofluorescence signal from unstained samples was obtained and subtracted for every sample. Data were analyzed using FlowJo v10 software.

### 4.11 SAβGal activity assay

SAβGal staining was performed using the Senescence β‐Galactosidase Staining Kit (Cell Signaling) following the manufacturer's instructions. Briefly, cells were fixed at room temperature for 2 min with a solution containing 2% formaldehyde and 0.2% glutaraldehyde in PBS, washed three times with PBS, and incubated overnight at 37°C with the Staining Solution containing X‐gal in DMF (pH 6.0).

### 4.12 Statistical methods

At least three independent replicates were assayed to ensure reproducibility in cell culture experiments. Statistical significance was assessed as appropriate using either the Student's *t*‐test (two‐tailed, paired, or unpaired), the Fisher exact test, or the one‐way ANOVA followed by a post hoc Dunnett's multiple comparisons test, as indicated in the figure legends. Data were tested for normal distribution using the Shapiro–Wilk test and for equal variance using the F‐test. Normal distribution and equal variance were confirmed in the majority of the data, and, therefore, normality and equal variance were assumed for all samples. Data were represented as the mean of the indicated experimental replicates. Error bars represent the standard deviation (SD) or the standard error of the mean (SEM), as indicated in the figure legends. *p* values inferior to 0.0001 were assigned a value of *p* = 0.0001. All *p* values are indicated in the figures.

## AUTHOR CONTRIBUTIONS

Miguel Rovira performed most of the experiments and contributed to experimental design, data analysis, discussion, and writing; Rebecca Sereda performed the studies with different pro‐senescent stimuli in NIH 3T3, Neuro2A, IMR90, and MEFs, prepared images of the reporters, and helped with proofreading; David Pladevall‐Morera performed the *RAB27A*‐KD experiments in SK‐MEL‐103 cells using shRNA and contributed to experimental design and discussion; Valentina Ramponi performed the experiments in IMR90 cells and studied the mRNA levels of lysosomal proteins; Ines Marin performed immunoblots of lysosomal proteins and performed the flow cytometry analysis of LAMP1 and LAMP2; Mate Maus performed immunoblots of lysosomal proteins; Julio Madrigal‐Matute helped with the lysosomal isolation experiments and contributed to experimental design and discussion; Antonio Díaz performed the total protein degradation assays, and the autophagy reporters experiments; Fernando García and Javier Muñoz performed the mass spectrometry analysis; Ana María Cuervo designed and supervised the lysosomal isolation experiments, the total protein degradation assays and the autophagy reporters experiments, and contributed to data analysis, discussion, and writing; Manuel Serrano designed and supervised the biological studies, analyzed the data, and wrote the manuscript. All authors revised and commented on the manuscript.

## FUNDING INFORMATION

M.R. was holder of a “la Caixa”‐Severo Ochoa PhD scholarship. J.M.‐M. was supported by a postdoctoral fellowship from the American Heart Association 17POST33650088a and R.S. was funded by a training grant T32GM007491. V.R. was funded by a PhD scholarship from the H2020‐MSCA‐ITN‐2018 HealthAge Program. I.M. received a PhD scholarship from the Spanish Ministry of Science (FPI Program). M.M. received funding from the European Union’s Horizon 2020 research and innovation programme under the Marie Sklodowska‐Curie grant agreement (No 794744) and from the Spanish Ministry of Science (RYC2020‐030652‐I /AEI /10.13039/501100011033). Work in the laboratory of A.M.C. was supported by National Institutes of Health grants AG021904, AG031782, and DK098408. Work in the laboratory of M.S. was funded by the IRB and “laCaixa” Foundation, and by grants from the Spanish Ministry of Science co‐funded by the European Regional Development Fund (ERDF) (SAF2017‐82613‐R), European Research Council (ERC‐2014‐AdG/669622), and Secretaria d'Universitats i Recerca del Departament d'Empresa i Coneixement of Catalonia (Grup de Recerca consolidat 2017 SGR 282). The funders had no role in data collection and analysis, decision to publish, or preparation of the manuscript.

## CONFLICT OF INTEREST

A.M.C. is co‐founder of Selphagy Therapeutics (now under LifeBiosciences, Boston, MA, USA) and consults for Generian Therapeutics and Cognition. M.S. is shareholder and advisor of Senolytic Therapeutics, Inc., Rejuveron Senescence Therapeutics, AG, and Altos Labs, Inc; and is shareholder of Life Biosciences, Inc. The funders had no role in study design, data collection and analysis, decision to publish, or preparation of the manuscript.

## Supporting information


Supplementary Figure 1
Supplementary Figure 2
Supplementary Figure 3
Supplementary Figure 4
Supplementary Figure 5
Supplementary Figure 6

Appendix S1

Appendix S2

Appendix S3

## Data Availability

The data that supports the findings of this study are available in the supplementary material of this article. The proteomic data are openly available in ProteomeXchange at [https://ebi.ac.uk/pride/] with identifier PXD022492.
