# Supplementary material for: The lysosomal proteome of senescent cells contributes to the senescence secretome
Source: Aging Cell. 2022 Sep 10;21(10):e13707. doi: 10.1111/acel.13707 (PMC9577959; doi:10.1111/acel.13707)
Supplement: Supplementary file 1 — Supplementary Figure 1 Supplementary Figure 2 Supplementary Figure 3 Supplementary Figure 4 Supplementary Figure 5 Supplementary Figure 6 [file ACEL-21-e13707-s002.pdf]

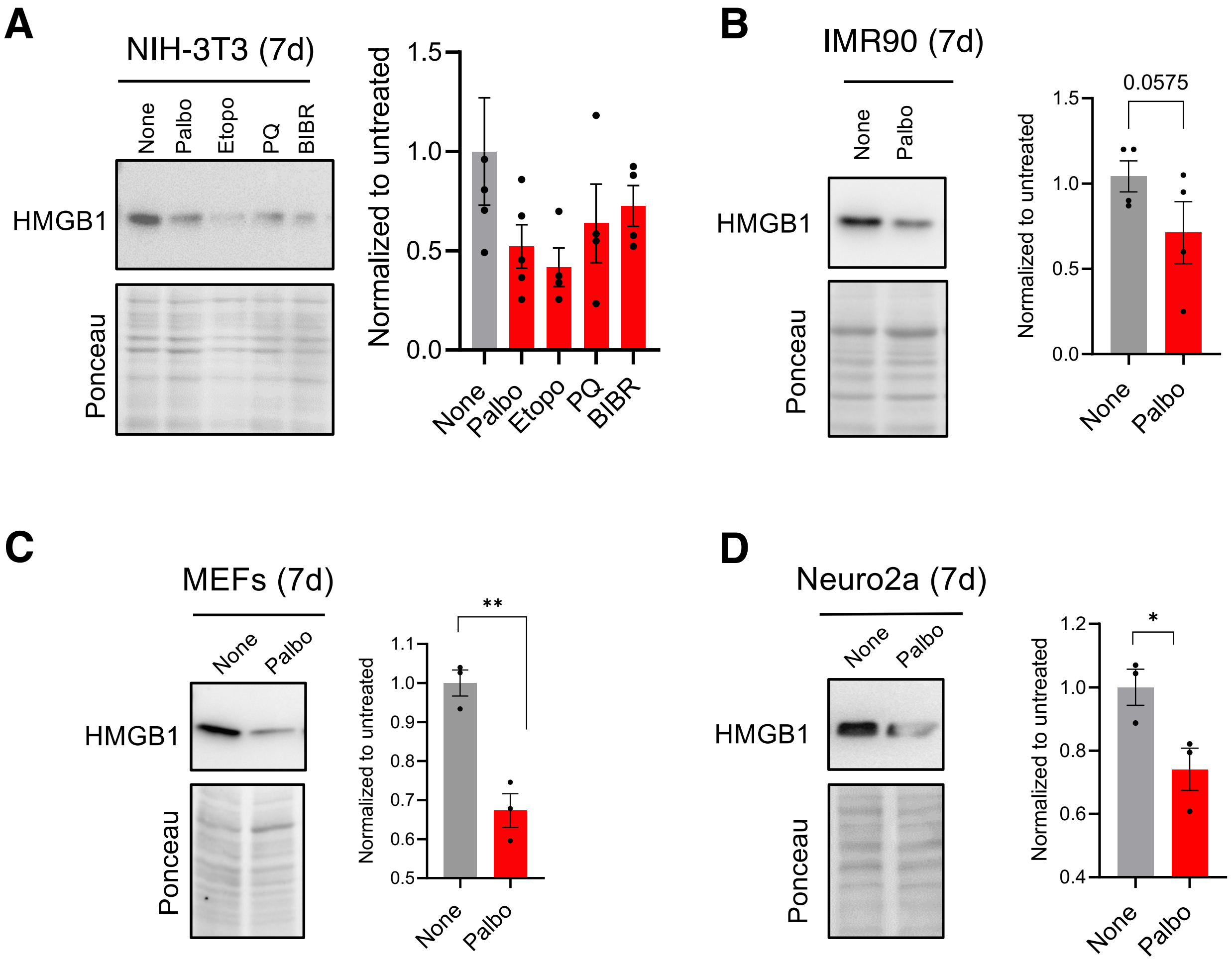

Supplementary Figure 1

Supplementary Figure 1.

A, *Left:* Representative immunoblot for HMGB1 of NIH3T3 cells untreated (None) or 7 days after exposure to the indicated agents. Ponceau staining is shown as loading control. *Right:* Quantification of HMGB1 levels from n = 4 independent experiments. Values are expressed relative to untreated cells and are individual values and mean ± SEM.

B, *Left:* Representative immunoblot for HMGB1 of human fibroblasts (IMR90 population doubling level (PDL) 20 untreated (None) or 7 days after exposure to palbociclib. Ponceau staining is shown as loading control. *Right:* Quantification of HMGB1 levels from n = 4 independent experiments. Values are expressed relative to untreated cells and are individual values and mean ± SEM of three independent experiments.

C, Similar to B using primary mouse embryonic fibroblasts (MEFs).

D, Similar to B using Neuro2a cells (N2a).

Statistical significance was estimated by 1-way ANOVA test (in A) and unpaired t-test in B-D, ** $p<0.01$, * $p<0.05$.

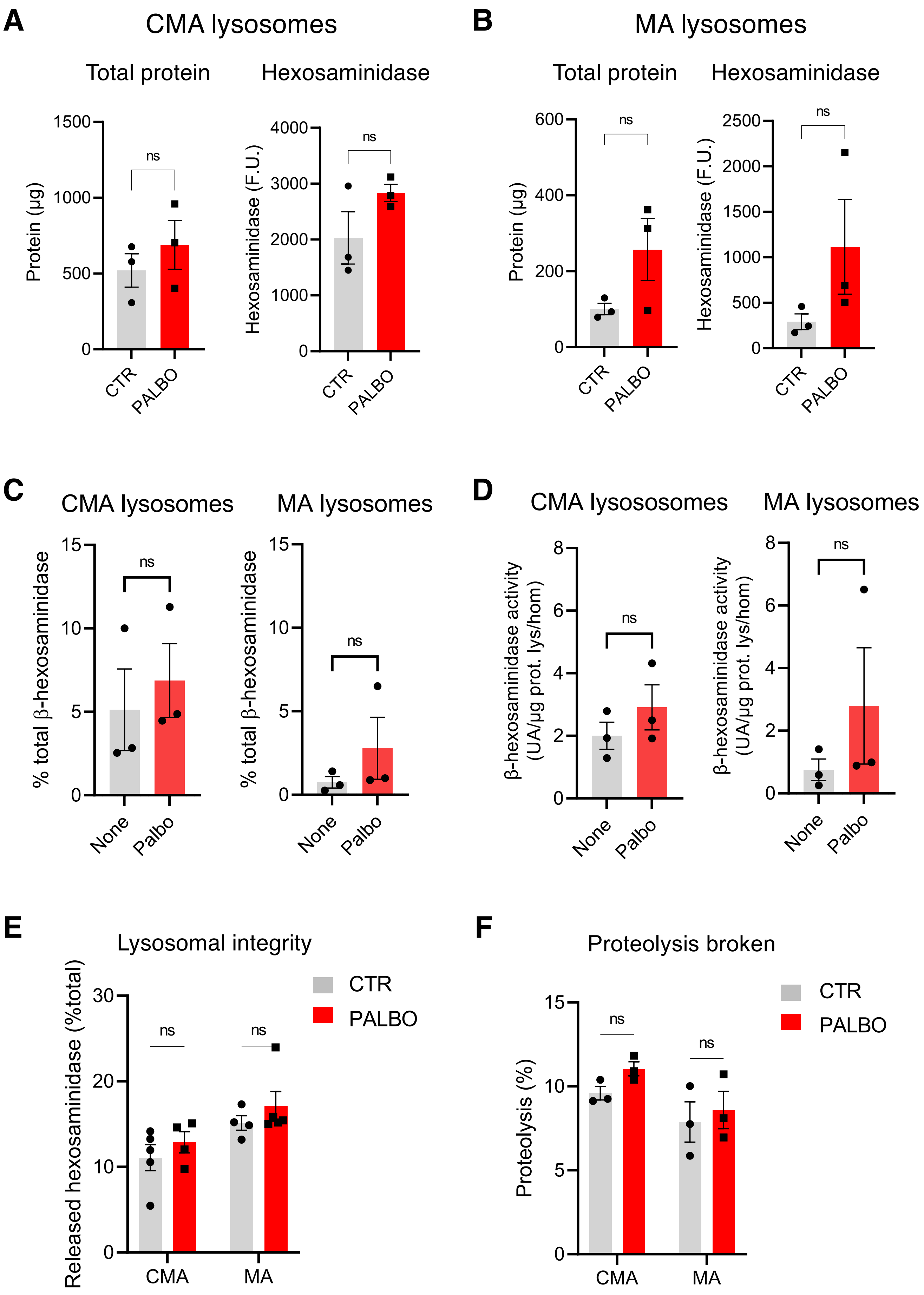

Supplementary Figure 2

Supplementary Figure 2.

A, Total protein (left) and hexosaminidase activity (right) in CMA lysosomes isolated from control or 7 days after palbociclib treatment of SK-MEL-103 cells. Values are individual values and mean ± SEM of three different isolations. FU: fluorescence units

B, Total protein (left) and hexosaminidase activity (right) in MA lysosomes isolated from control or 7 days palbociclib-treated SK-MEL-103 cells. Values are individual values and mean ± SEM of three different isolations. FU: fluorescence units

C, Percentage of total cellular hexosaminidase activity recovered in CMA (left) and MA (right) lysosomes isolated as in A and B. Values are individual values and mean ± SEM of three different isolations.

D, Enrichment for hexosaminidase activity in CMA (left) and MA (right) lysosomes isolated as in A and B calculated as units of activity (UA) per μg of protein in the fractions relative to activity in the homogenate. Values are individual values and mean ± SEM of three different isolations.

E, Lysosomal membrane stability measured as the percentage of lysosomal β-hexosaminidase activity detectable in the media relative to total β-hexosaminidase in the lysosomal preparations. Values are individual values and mean ± SD, n=4.

F, Proteolytic capacity of luminal content of CMA and MA lysosomes isolated from control or 7 days palbociclib-treated SK-MEL-103 cells. Values are individual values and mean ± s.e.m, n=3.

Statistical significance was estimated by unpaired t-test (A-D) and two-way ANOVA and Sidak's multiple comparisons test (E, F). n.s.: non-significant.

A

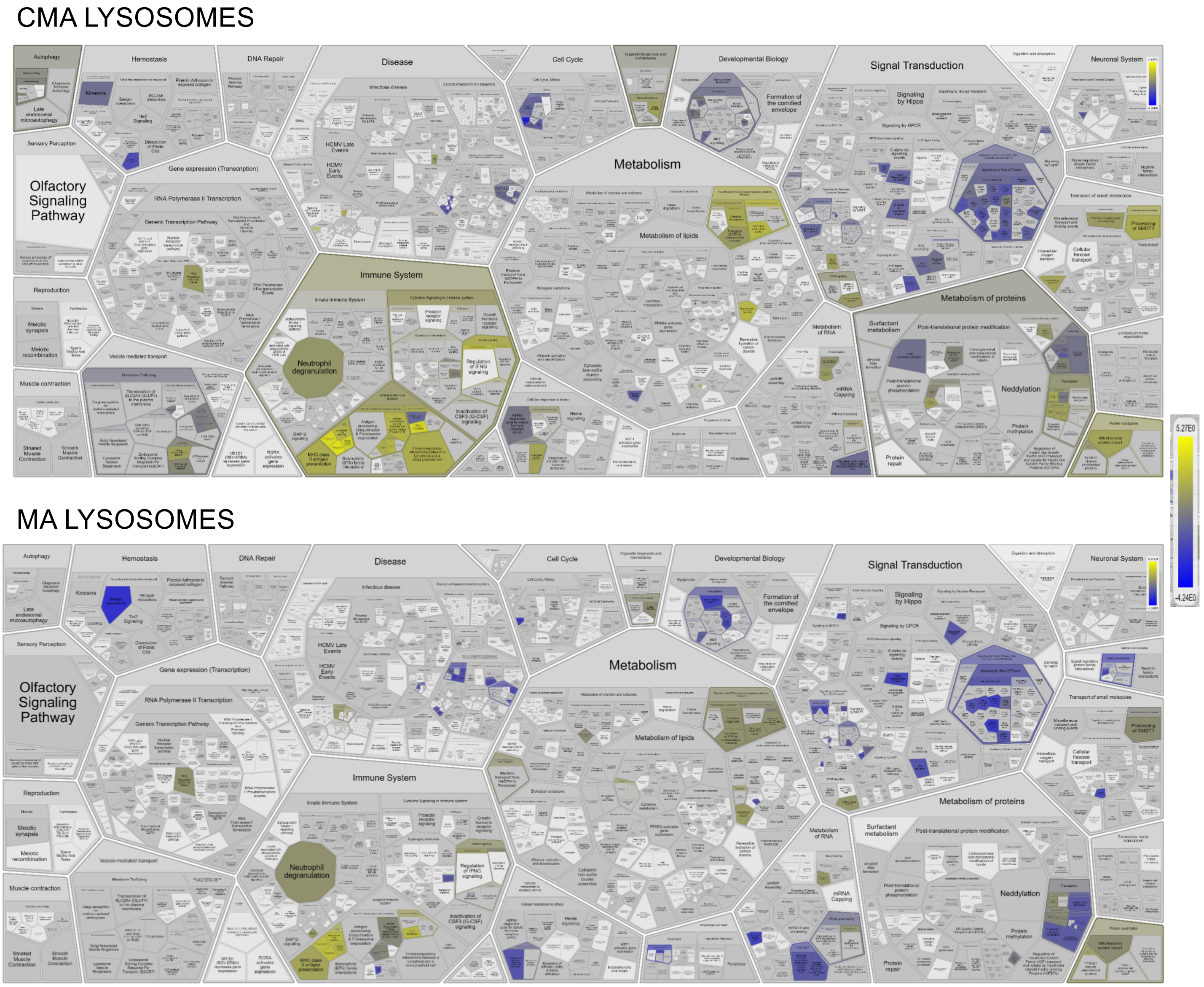

B

CHANGES IN CMA LYSOSOMES ONLY

↑ Iron uptake/transport
Cargo concentration in ER
COPI-mediated transport
Transferrin endocytosis

↓ Rab-generylation
Kinesins
Intra Golgi trafficking

CHANGES IN MA LYSOSOMES ONLY

↑ Cell junction organization

↓ Strogen-stimulated signals
Cell junction adherines
Apoptotic proteins
Reeling and Robo

CHANGES IN CMA AND MA LYSOSOMES

↑ mTOR signaling
TCR donswstream
MHC class II antigen presentation
PD-1 signaling

↓ RhoGTPases
CTL4 inhibitory signaling

Supplementary Figure 3

Supplementary Figure 3.

A, Mosaic gradient representation of the Reactome analysis of proteins with increased (yellow) or reduced (blue) abundance in CMA active lysosomes (top) and MA lysosomes (bottom) in 7 days palbociclib-treated cells SK-MEL-103 cells compared to control cells.

B, Cellular pathways of proteins that change in senescent SK-MEL-103 cells compared with control only in CMA lysosomes, only in MA lysosomes or that change in both. Arrow indicates decreased (blue) or increased (orange) levels in senescent cells.

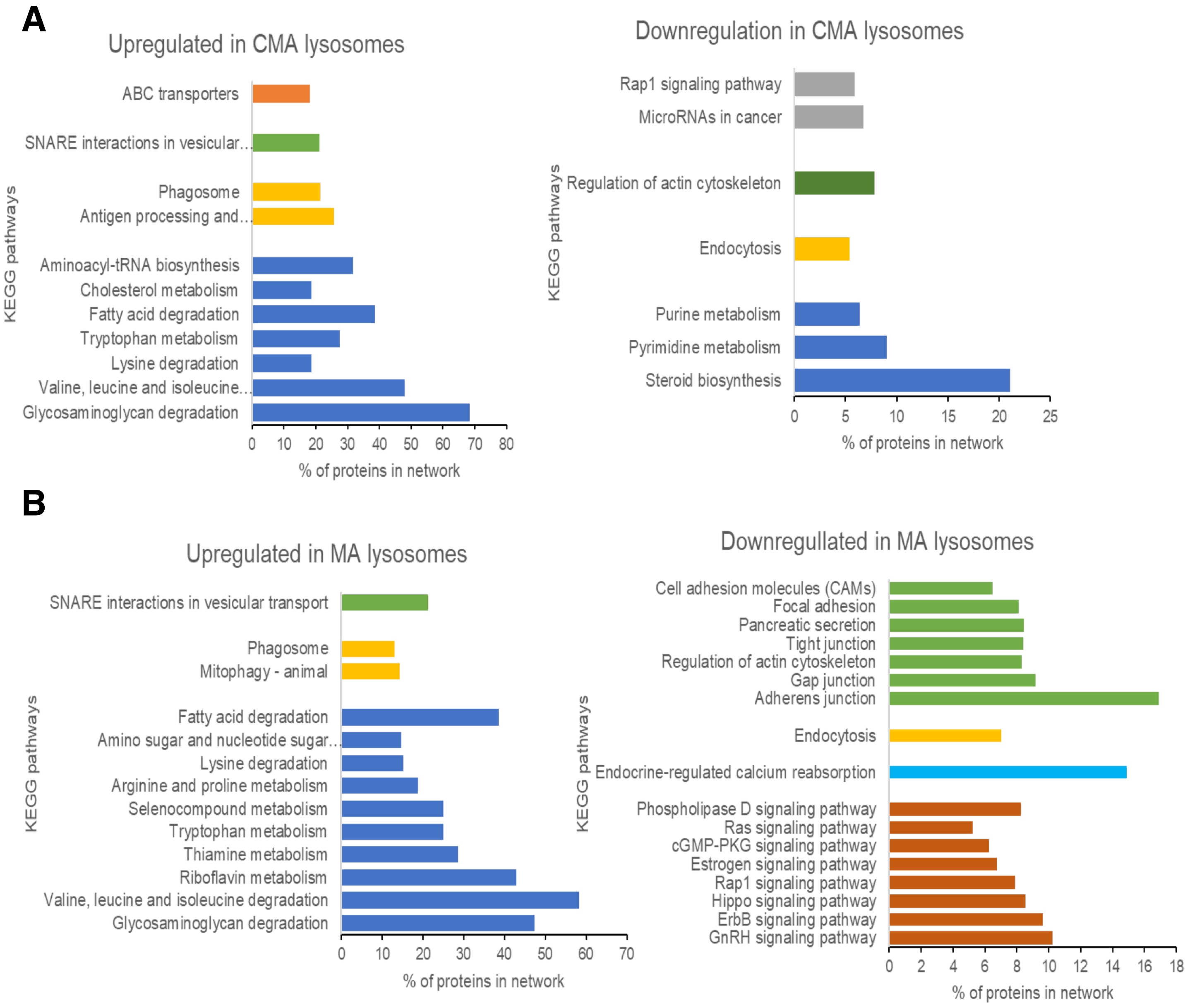

Supplementary Figure 4

Supplementary Figure 4.

A, GO terms gene sets of proteins defined as resident lysosomal proteins that show higher (left) or lower (right) levels in CMA lysosomes from SK-MEL-103 senescent cells. Constitutive resident proteins were defined as those that do not change significantly $p > 0.05$ in N/L *vs* vehicle or that have a negative log2 fold change N/L *vs* vehicle.

B, GO terms gene sets of proteins defined as resident lysosomal proteins that show higher (left) or lower (right) levels in MA lysosomes from SK-MEL-103 senescent cells. Constitutive resident proteins were defined as those that do not change significantly $p > 0.05$ in N/L *vs* vehicle or that have a negative log2 fold change N/L *vs* vehicle.

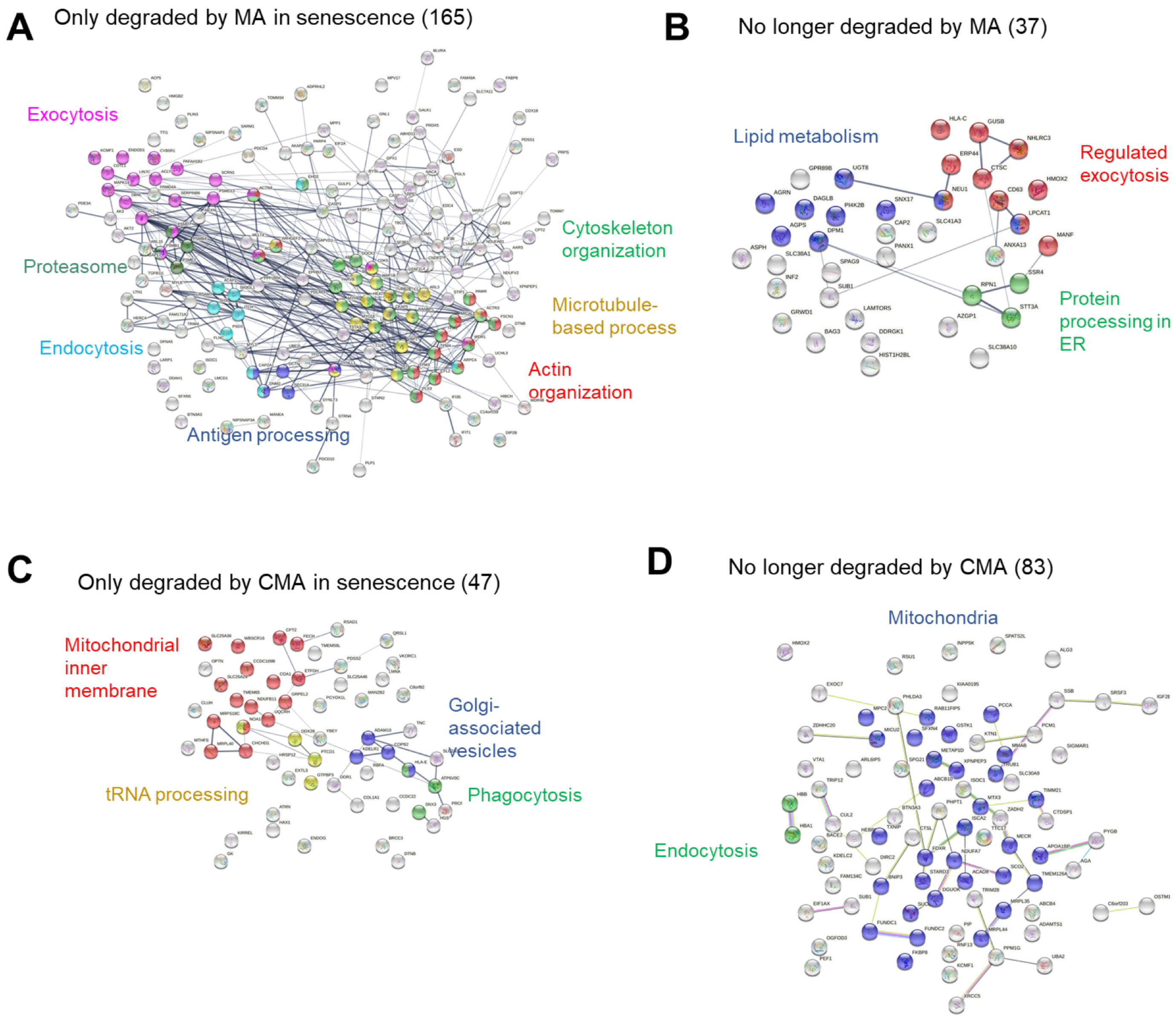

Supplementary Figure 5

Supplementary Figure 5.

A, B, STRING analysis of proteins identified in the proteomic analysis of lysosomes isolated from control or 7 days palbociclib-treated SK-MEL-103 cells that are only degraded (**A**) or no longer degraded (**B**) by MA in senescent cells. Substrate proteins were defined as those that accumulate significantly upon N/L treatment, *i.e.,* log2 fold change N/L *vs* vehicle >0.21 (fold >1.1) and p value <0.05.

C, D, STRING analysis of proteins identified in the proteomic analysis of lysosomes isolated from control or 7 days palbociclib-treated SK-MEL-103 cells that are only degraded (**C**) or no longer degraded (**D**) by CMA in senescent cells. Substrate proteins were defined as those that accumulate significantly upon N/L treatment, *i.e.,* log2 fold change N/L *vs* vehicle >0.21 (fold >1.1) and p value <0.05.

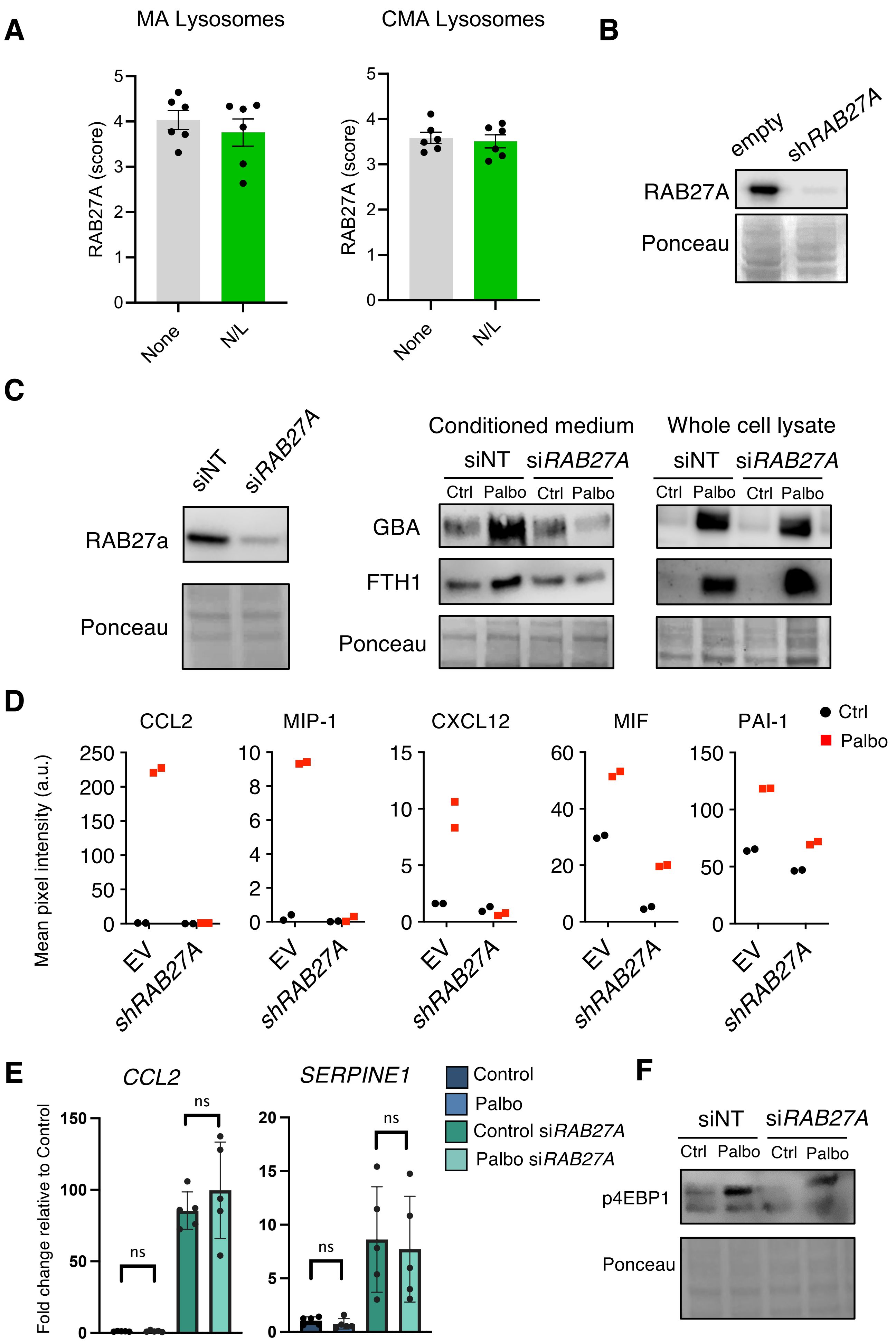

Supplementary Figure 6

Supplementary Figure 6.

A, Levels of RAB27A in MA (left) and CMA (right) lysosomes isolated from SK-MEL-103 cells cultured without additions (none) or in the presence of 10 mM ammonium chloride and 100 μM leupeptin for 16h before isolation. Individual values and mean ± SEM from three different isolations in duplicate are shown. Values are expressed as Z score were extracted from the MS proteomic analysis.

B, Confirmation of the knock-down of RAB27A using lentiviral shRNA or empty vector in SK-MEL-103 cells.

C, Confirmation of the knock-down of RAB27A using siRNAs in SK-MEL-103 cells (left). Analysis of the levels of GBA and FTH1 in SK-MEL-103 cells, control or senescent, treated or not with siRNAs targeting RAB27A (right). Protein levels were analyzed in the conditioned medium and in whole-cell extracts

D, Profiles of mean spot pixel density for the cytokines and chemokines indicated in Fig. 7E. EV, empty vector; sh, lentiviral sh*RAB27A*.

E, Levels of mRNA for the indicated genes in SK-MEL-103 cells, control or senescent (palbociclib), treated with non-targeting siRNAs (siNT) or with siRNAs targeting *RAB27A* (si*RAB27A*).

F, Levels of phospho-4EBP1 in the same cells as in panel E.

Statistical significance was estimated by t-test, n.s.: non-significant.
